# The *Entamoeba histolytica*, Arp2/3 Complex Is Recruited to Phagocytic Cups through an Atypical Kinase EhAK1

**DOI:** 10.1371/journal.ppat.1005310

**Published:** 2015-12-08

**Authors:** Mrigya Babuta, M Shahid Mansuri, Sudha Bhattacharya, Alok Bhattacharya

**Affiliations:** 1 School of Life Sciences, Jawaharlal Nehru University, New Delhi, India; 2 School of Environmental Sciences, Jawaharlal Nehru University, New Delhi, India; 3 School of Natural Sciences, Department of life Sciences, Shiv Nadar University, Uttar Pradesh, India; University of Virginia Health System, UNITED STATES

## Abstract

The parasite *Entamoeba histolytica* is the etiological agent of amoebiasis and phagocytosis plays a key role in virulence of this organism. Signaling pathways involved in activation of cytoskeletal dynamics required for phagocytosis remain to be elucidated. Phagocytosis is initiated with sequential recruitment of EhC2PK, EhCaBP1, EhCaBP3 and an atypical kinase EhAK1 after particle attachment. Here we show that EhARPC1, an essential subunit of the actin branching complex Arp 2/3 is recruited to the phagocytic initiation sites by EhAK1. Imaging, expression knockdown of different molecules and pull down experiments suggest that EhARPC1 interacts with EhAK1 and that it is required during initiation of phagocytosis and phagosome formation. Moreover, recruitment of EhARPC2 at the phagocytosis initiation by EhAK1 is also observed, indicating that the Arp 2/3 complex is recruited. In conclusion, these results suggests a novel mechanism of recruitment of Arp 2/3 complex during phagocytosis in *E*. *histolytica*.

## Author Summary

*E*. *histolytica* is the causative agent of amoebiasis and leads to morbidity and mortality in developing countries. It is known to phagocytose immune and non-immune cells, epithelial tissue, erythrocytes and commensal bacteria. The high rate of phagocytosis in this protist parasite provides a unique system to study the signaling cascade that is activated after attachment of the particle to the cell surface. The major objective of the signaling pathway is to generate force for uptake of the particle and this is done through stimulating cytoskeleton to form appropriate structures. However, the molecular mechanism of the same is still largely unknown in *E*. *histolytica*, though this pathway has been characterized in many other systems. We have been investigating this pathway by using red blood cells as a particle and have identified different molecules required during the initial stages of phagocytosis. In this study we demonstrate the mechanism by which actin cytoskeleton branching complex EhARP2/3 is recruited at the site of erythrophagocytosis and show that the recruitment is through an atypical alpha kinase EhAK1. A number of different approaches, such as pull down assay, conditional suppression of EhAK1 expression and imaging were used to decipher this pathway. Therefore this study provides a mechanism by which actin dynamics couples to the initial signaling system, activated on attachment of RBC to the cell receptors.

## Introduction

Phagocytosis plays a critical role in invasion and pathogenesis of the parasite *Entamoeba histolytica*, the causative agent of amoebiasis, and a major cause of morbidity and mortality in developing countries. Phagocytosis is an essential process in *E*. *histolytica* as blocking this process leads to an inhibition of cell proliferation and pathogenicity [1, 2].

The pathways coupling phagocytosis initiation signals to actin dynamics have been studied quite extensively in some model systems [3–5]. In mammalian and other systems a number of proteins that bind and regulate actin nucleation, polymerization, bundling, and branching have been identified and characterized. Arp2/3 complex is one of the main group of molecules required for actin dynamics. It comprises of seven subunits, Actin related protein 2 (Arp2, 44KDa), Actin related protein 3 (Arp3, 47KDa), ARPC1 (40KDa), ARPC2 (35KDa), ARPC3 (21KDa), ARPC4 (20KDa) and ARPC5 (16KDa). There are multiple ways by which Arp 2/3 complex is recruited at the site of actin dynamics. Some of the examples are, interaction with VCA domain of activated NPFs (nucleation protein factors) [6], direct binding of Arp2/3 complex to vinculin (an integrin associated protein) during cell migration at the sites of integrin-mediated adhesions and membrane protrusions and binding of F-actin [7], direct binding to cortical actin associated protein (cortactin) [8] and recruitment through WAVE2 complex during T-cell activation [9]. Though NPFs, such as WAVE, WASP and WASH are thought to be involved in activation of Arp 2/3 complex during phagocytosis, in some situations (e.g. uptake of *Yersinia pseudoparatuberculosis*) the pathway through N-WASP is by passed and Arp 2/3 complex is directly activated by Rac-1[10]. We can conclude from this discussion that there are multiple ways by which Arp 2/3 complex gets activated in different systems.

However, there is not much information available about the pathway(s) and regulatory steps during phagocytosis in *E*. *histolytica*. Two approaches were used to understand the mechanism of phagocytosis in *E*. *histolytica*. In one approach sequence similarity searches were used to identify putative homologous proteins that are known to participate in phagocytosis in other systems [11]. In the second approach, phagosome proteome of *E*. *histolytica* was analysed using mass spectrometry [12–14]. A summary of the published results is shown in the S1 Table. Sequence similarity analysis identified all subunits of Arp 2/3 complex in *E*. *histolytica*, and a few of the proteins known to be involved in recruitment and activation of this complex during actin dynamics [11]. Some of these are; a homolog of WASP protein containing a conserved VCA domain, a homolog of MIM which also contains a VCA domain, eight formins, filamins and alpha actinins. Not all of these proteins were consistently found in phagosome proteome. For example, WASH homolog was identified in only one of the experiments. Moreover, it is not clear if these homologs carry out the same function in *E*. *histolytica* as is known as in other systems, since experimental evidence to this effect is still not available in *E*. *histolytica*. Since many proteins involved in initiation or scission of phagosomes are lost either before or soon after phagosome formation (for example EhCaBP1 [15]), the lack of participation of a molecule in phagocytosis cannot be inferred based only on its absence in phagosome proteome.

Some of the actin modulating proteins have been studied at functional level in *E*. *histolytica*. Foremost among these are Formins. Formin 1 and 2 were found to colocalize with actin in pseudopodia, cell division sites and in both pinocytic and phagocytic vesicles suggesting that these may be involved in cell division, pinocytosis and phagocytosis [16]. EhFormin1 was also shown to modulate actin polymerization through its formin homology 2 domain. EhRho1 is thought to regulate directly activating this protein [17]. In general coactosins belong to ADF/cofilin family and are known as F-actin severing and depolymerising proteins [18]. On the contrary, Ehcoactosin was found to stabilize F-actin [19]. EhFLN (previously known as EhABP-120), a filamin protein is recruited at the plasma membrane via PI(3)P and phosphatidic acid (PA). When the d100 region of EhFLN required for binding to PA, was over-expressed it increased the amoebic motility suggesting its role in actin dynamics [20]. In addition other actin binding proteins such as 16 kDa and EhNCABP166 have been partially characterized. On down regulation of expression of the former, an inhibition in cell motility was observed [21]. EhNCABP166 is present in both cytosol and nucleus and is thought to be involved in phagocytosis and cell motility [22, 23]. It is clear from the discussion that there are a number of likely components of actin dynamics machinery. However, the participation of specific components in different cellular processes has not been worked out in detail.

We have been investigating molecular mechanisms that are involved in the initiation of phagocytosis using red blood cell (RBC) uptake as a system. Our major effort has been to identify molecules that are needed for initiation of a protein complex at the site of particle attachment leading to phagocytic cup formation, and channeling actin dynamics for progression of phagocytic cups to phagosomes. The nature of the primary signal generated after the attachment has not been elucidated so far, though there is some evidence to suggest that the GPI anchor present in Gal/GalNAc lectin complex may be the key transducing component [24, 25]. Some of the other proteins shown to be involved in phagocytosis in *E*. *histolytica* are phagosome-associated transmembrane kinase [26], serine-rich *E*. *histolytica* protein [27], EhPAK [28], and EhCaBP5 [29]. It is not clear how and in which stages these molecules participate in the phagocytic process. For example, cell surface molecules PATMK and SREHP are suggested to be involved in erythrophagocytosis but it is not clear whether they are the receptor for particles, or are the initiator molecules required for transducing signal immediately after particle attachment.

Our studies have shown that the primary signal helps to enrich a C2 domain protein kinase, EhC2PK, at RBC-attachment sites [30]. EhC2PK recruits calcium binding protein EhCaBP1 [15, 30], which in turn brings in atypical kinase EhAK1 at the site of attachment [31]. Another calcium binding protein EhCaBP3 is independently recruited forming a multimeric complex [32]. All these proteins have different roles during progression of phagocytic cups to phagosomes. While both EhC2PK and EhCaBP1 leave phagocytic cups before closure of cups, EhAK1 is found in just closed cups before scission and only EhCaBP3 is present in the phagosomes after scission (mature phagosomes) [15, 30–32]. Nearly twenty proteins were found to interact with EhCaBP1 as determined by affinity chromatography and mass spectrometry [30]. Among these only EhARPC1, a member of the Arp2/3 complex, was found to be a potential molecule that may couple EhCaBP1-EhC2PK mediated signaling with actin dynamics. Arp2/3 complex proteins EhARPC1 and EhARPC2 were also found when EhAK1 binding proteins were analyzed by mass spectrometry [31]. Absence of other actin modulating proteins in these two pull down experiments suggests that EhARPC1 and EhARPC2 may have important role in the phagocytic pathway mediated by EhCaBP1-EhC2PK.

In this report we describe the role of EhARPC1, one of the subunits of Arp2/3 complex, in the phagocytosis of RBC in *E*. *histolytica*. Our results show that it is recruited to the phagocytic cups through EhAK1 and participates in phagocytosis. We also show that another subunit of Arp 2/3 complex EhARPC2 is recruited at the cups, suggesting the presence of Arp 2/3 complex at the phagocytic site. Our results provide a basis for coupling of actin dynamics to the initial signaling system activated on attachment of a RBC to the cell membrane. The pathway described by us is novel and has not been seen in any other system so far.

## Results

### EhARPC1 is a conserved protein with WD40 repeats

Sequence analysis of EhARPC1 showed maximum identity with p41 subunit of Arp2/3 complex from a number of species. Identity ranged from 32% with *Saccharomyces cerevisiae* to 41% with human. Multiple alignment of amino acid sequences of homologs from different species displayed conservation across the full length of the protein, higher towards N-terminal region than C-terminal (S1A Fig). The p41 subunit of Arp 2/3 complex of all organisms contain conserved WD40 repeats. Therefore, it was not surprising to find probable WD40 repeats (amino acids 50 to 181) in the amoebic protein as well. However, the WD40 repeat containing region in the amoebic protein was longer than the corresponding region of human protein (48 to 89 amino acids) or yeast (51 to 92 amino acids) but was similar to that of *Dictyostelium discoideum* protein (50–180 amino acids) (S1B Fig). Moreover, “Arm” region (C-terminal sequence) in p41 subunit of *S*. *cerevisiae* that is required for binding WASP, is absent in EhARPC1 [33] (S1C Fig). It appears from sequence analysis that EhARPC1 may have diverged functionally from the human or the yeast homologs.

Arp2/3 complex comprises of seven subunits and EhARPC1 is one of its members, as mentioned before sequence analysis has suggested presence of all seven subunits in *E*.*histolytica* only EhArp2 and EhArp3 showed maximum identity with Arp2 and Arp3 of *D*. *discoideum* and EhARPC5,was found to be maximally diverged from that of other species (S2 Table).

### Cellular localization of EhARPC1

**Fig 1:**
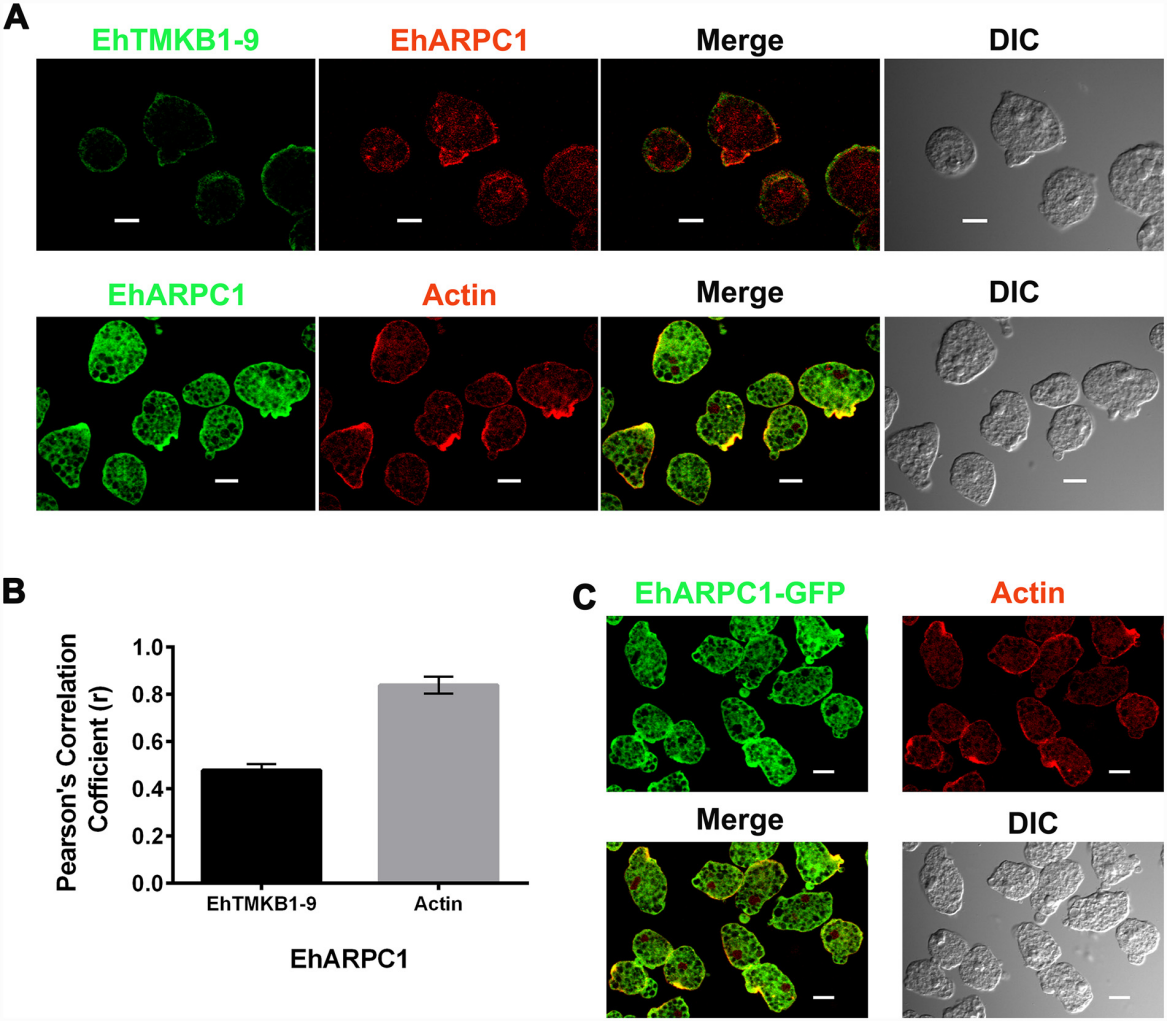
Localization of EhARPC1 in *E*. *histolytica* trophozoites. **(A)** *E*. *histolytica* cells (without RBC and other cells) were stained for EhARPC1, EhTMKB1-9 (as membrane marker) and actin using mouse anti-EhARPC1 antibody, rabbit anti-EhTMKB1-9 antibody and TRITC conjugated phalloidin respectively, followed by appropriate second antibody labeled with Alexa488 or Alexa-555. **(B)** Quantitative analysis of colocalisation of EhARPC1 with either EhTMKB1-9 or actin based on ten stained images as in (a) was carried out using Pearson’s correlation coefficient (r). **(C)** Immunolocalization of GFP-tagged EhARPC1 with respect to actin. Cells expressing GFP-EhARPC1 were harvested and fixed followed by staining with anti-GFP antibody and appropriate secondary antibody conjugated with Alexa 488. Actin was stained using TRITC phalloidin. (Scale bar, 10μm; DIC, differential interference contrast).

We carried out immunofluorescence imaging for determining cellular distribution of EhARPC1 using antibody raised against recombinant protein (specificity of the antibody is shown in S2A Fig). As a membrane marker, antibody against the *E*. *histolytica* pan membrane marker EhTMKB1-9 was used [34]. F-actin was visualised using TRITC-phalloidin. We found EhARPC1 in the cytoplasm, some parts of the membrane and in F-actin rich areas (Fig 1A). In order to investigate if EhARPC1 accumulates in some sites more than others, a quantitative analysis of the images was carried out. We estimated strengths of co-localization using fluorescent signals of a pair of stains analysed using Pearson’s correlation coefficient (PCC) (Fig 1B). We found preferential localization of EhARPC1 in actin rich areas (r = 0.898) and much less in plasma membrane (r = 0.483). A considerable amount of fluorescent signal of EhARPC1 originated from the cytoplasm. The results suggest that EhARPC1 is likely to be a cytoplasmic protein that gets recruited at F-actin-rich sites. Localization of Arp2/3 complex in model systems has also revealed preferential recruitment at the leading edges of lamellipodia in mammalian cells [35] and at the actin patches in *S*. *cerevisiae* [36]. The association of EhARPC1 with actin was further validated using *E*. *histolytica* cells expressing GFP-EhARPC1. The distribution of GFP fluorescence was similar to that seen by antibody staining (Fig 1C). Fluorescence signals strongly localized with F-actin enriched areas (r = 0.886), and a significant amount of fluorescence came from the cytoplasm as revealed by analysis of distribution across the whole cell (S2B and S2C Fig). The results suggest that GFP tagged protein behaved in the same way as native protein, residing mainly in cytoplasm but getting recruited to F-actin rich areas (Fig 1C).

### EhARPC1 is involved in pseudopod formation and phagocytosis

**Fig 2:**
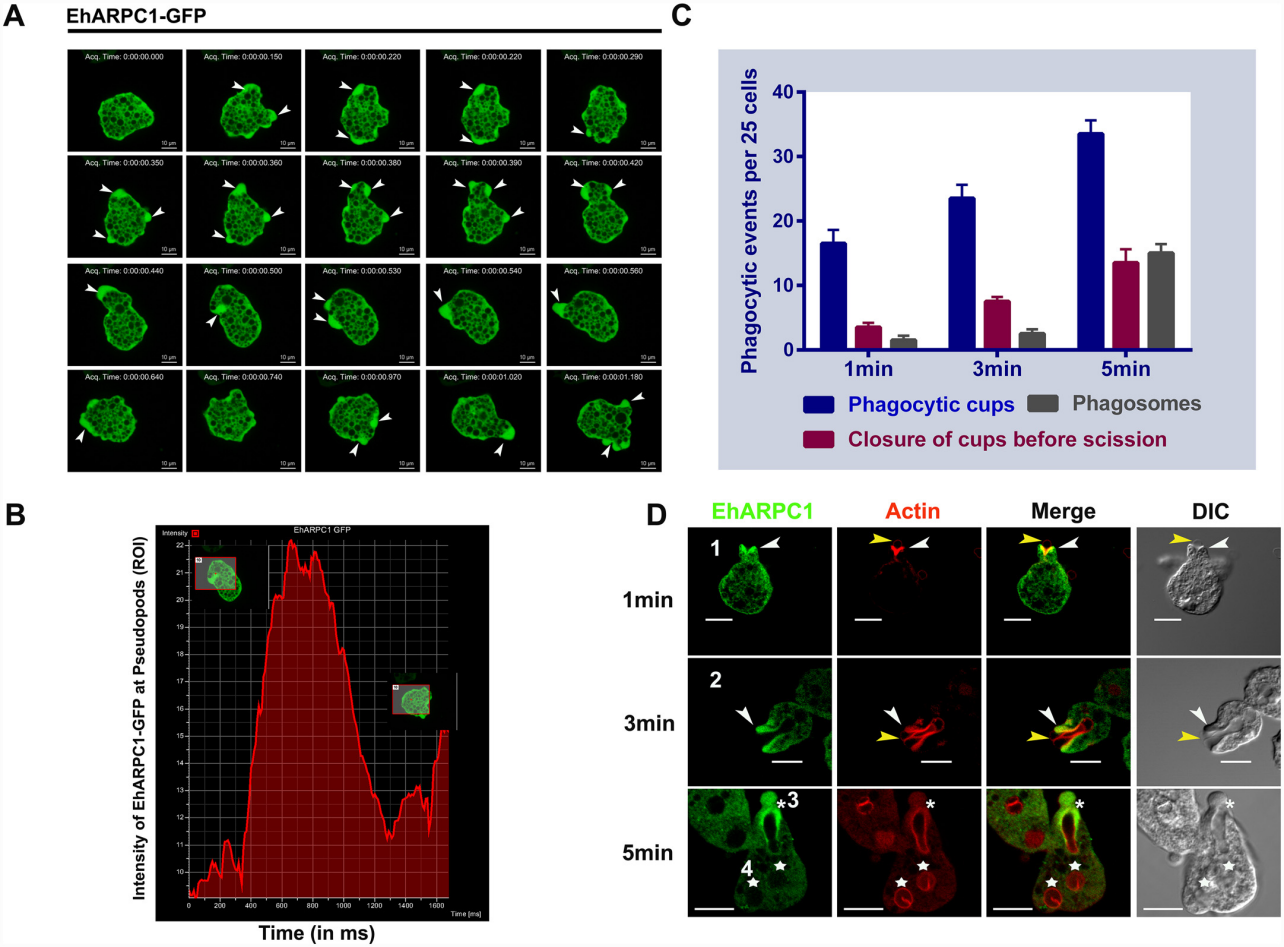
Involvement of EhARPC1 in pseudopod formation and phagocytosis. **(A)** The montage shows a time series of motile trophozoites expressing GFP-EhARPC1. A number of pseudopods in different directions can be visualized and are marked by white arrowheads. Bar represents 10μm. **(B)** Time course of intensity of GFP-EhARPC1 (ROI) was determined at leading edge of amoeba. Snapshot of ROI selected is shown at two time points where GFP-EhARPC1 fluorescent intensity increases and then gradually decreases. **(C)** Quantitative determination of phagocytic events was carried out in 25 cells by randomly selecting them and counting the number of phagocytic cups, closed cups before scission, and phagosomes present in these cells. **(D)** *E*. *histolytica* trophozoites actively phagocytosing RBCs, incubated for different times and representing different stages of phagocytosis were stained for EhARPC1 (Alexa 488) and actin (TRITC phalloidin). Arrowhead indicate phagocytic cups, asterisks mark closure of cup before scission, stars mark phagosomes, and yellow arrowheads indicate RBCs in the process of phagocytosis. Bar represents 10μm.

Binding of phagocytic ligands to the cell surface, triggers directed reorganization of cytoskeleton underneath the binding site, resulting in pseudopod formation. Pseudopods extend around the particle, fuse and then separate out from the membrane to form phagosomes. In order to investigate if EhARPC1 may be involved in pseudopod, and subsequent phagosome formation, we first measured enrichment of EhARPC1 at the pseudopods by live cell imaging of *E*. *histolytica* trophozoites expressing GFP-EhARPC1. We clearly saw enrichment of EhARPC1 at the moving (or leading) edge of amoebae, marked by arrowhead in images (Fig 2A and S1 Movie). This was confirmed by quantitative analysis of the images (Fig 2B). The time taken to complete a psuedopod formation and retraction was found to be 90ms± 20ms, indicating that the process is extremely rapid.

We further investigated the involvement of EhARPC1 in phagocytosis using a number of different approaches. Imaging was used to localize EhARPC1 during uptake of RBCs by *E*. *histolytica*. Cells were incubated for different times with RBCs, followed by fixing and staining with indicated antibodies. Images were analysed by quantifying the events in 25 cells (Fig 2C). The panel in Fig 2D shows representative images of cells displaying different stages of phagocytosis, such as early phagocytic cup (marked by 1), late phagocytic cup (marked by 2), closure of cups before scission (marked by 3) and mature phagosome (marked by 4). It is clear from the figure that EhARPC1 is recruited early after initiation of phagocytic cups and it stays till cups close, but have not yet undergone scission. It is not present in phagosomes, that is, it leaves during the process of scission (Fig 2D). Similar patterns were visualized when experiments were carried out using fluorescent labeled RBCs and GFP-EhARPC1 (S2D and S2E Fig respectively).

**Fig 3:**
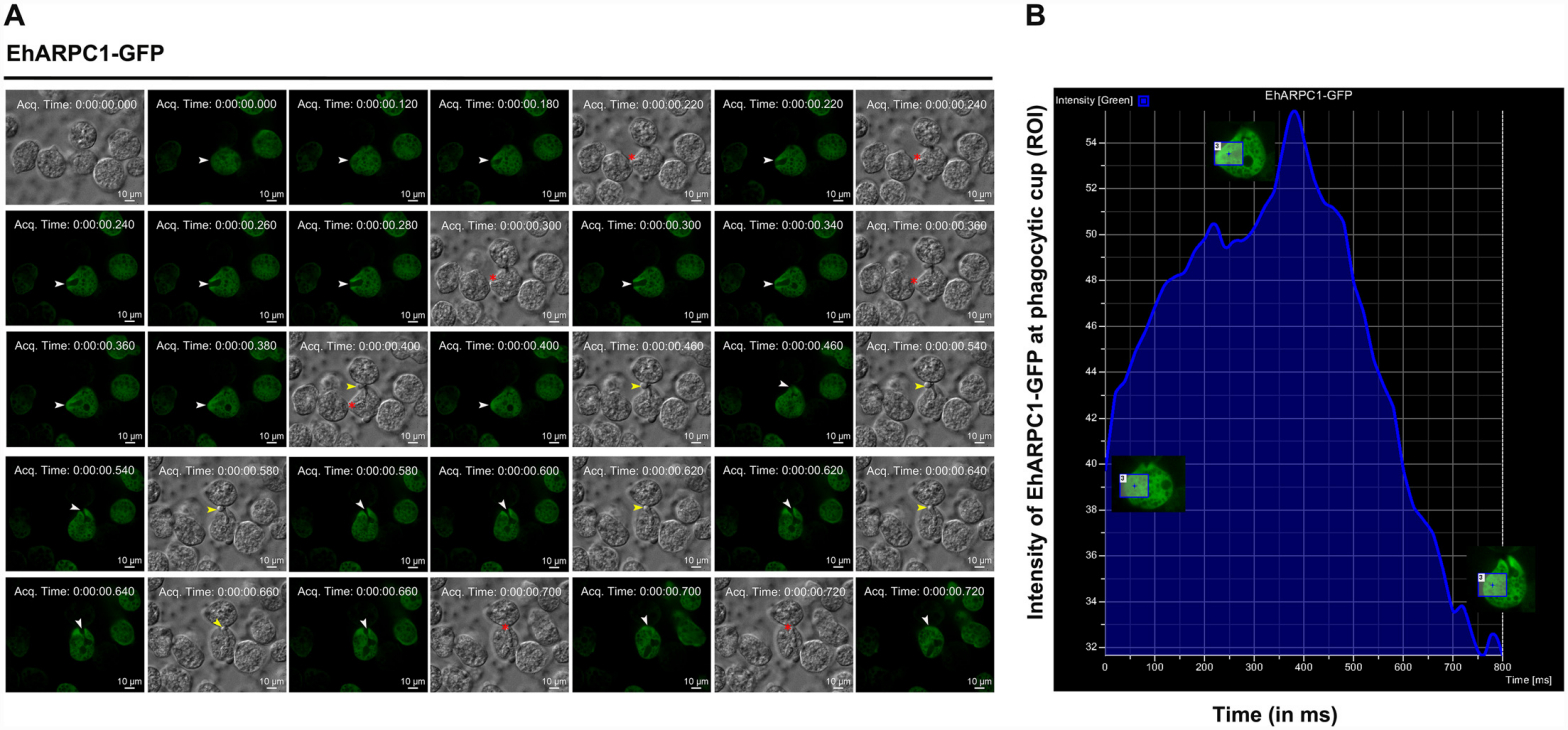
Localization of EhARPC1-GFP in phagocytosing RBC’s. **(A)** The montage shows a time series of GFP-EhARPC1 expressing cells undergoing erythrophagocytosis. Phagocytic cups are marked by arrowheads; RBCs in DIC are marked by yellow color arrowhead and phagocytosed RBCs by red asterisk. Bar represents 10μm. **(B)** Graph shows the intensity of GFP-EhARPC1 with respect to ROI during phagocytosis. Images of cells at different time points are shown in a box.

We also visualized RBC uptake in GFP-EhARPC1 expressing cells by time lapse imaging (S2 Movie). Generally, a cycle of phagocytosis is completed within 240-260ms after attachment of RBC. The data is shown as snap shots of a complete cycle at intervals (Fig 3A). Quantitative analysis of the data is shown in Fig 3B. GFP-EhARPC1 was present during cup formation and was found till the process of scission starts. It was not found in early phagosomes. We could clearly observe RBC attached to the surface of *E*. *histolytica* cells (marked by a yellow colored arrow in DIC images).

**Fig 4:**
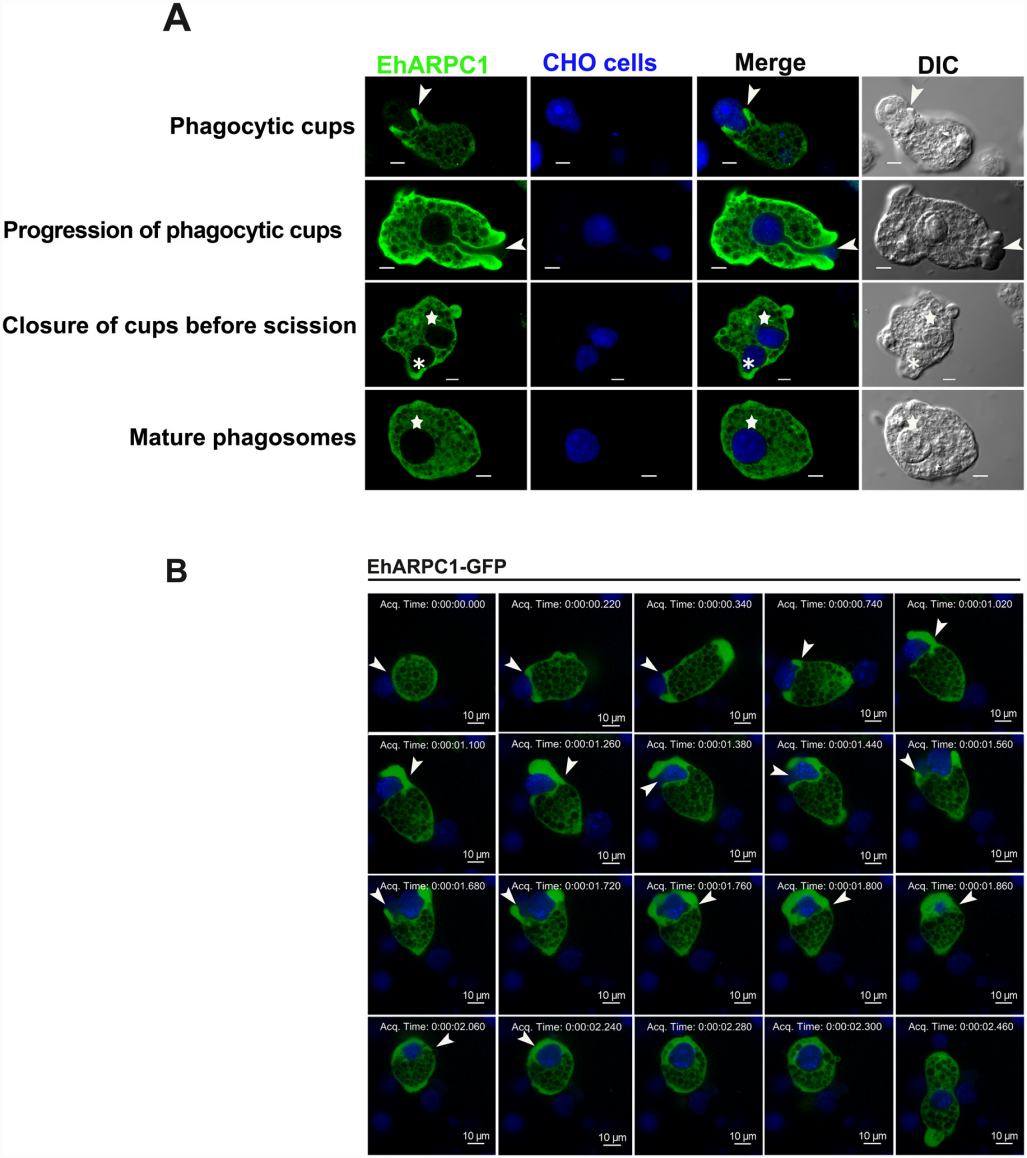
Localization of EhARPC1 during phagocytosis of CHO cells. **(A)** *E*. *histolytica* trophozoites were first incubated with cell tracker blue dye-labelled live CHO cells for different times, and then were fixed and stained for EhARPC1 (Alexa 488). Arrowhead indicate phagocytic cups, asterisks mark closure of cups before scission and star marks phagosomes. Scale Bar represents 10 μm. **(B)** The montage shows *in vivo* localization of GFP-EhARPC1 during the uptake of CHO cells, where CHO cells are stained with Cell tracker blue dye. Bar represents 10μm.

EhARPC1 was also found to involve in phagocytosis of other ligands, such as mammalian cells. This was visualized by observing enrichment of EhARPC1 during phagocytosis of Chinese hamster ovary cells (CHO) labeled with cell tracker blue dye (Fig 4A), and by time lapse imaging (snap shots Fig 4B and S3 Movie). EhARPC1 was observed at the phagocytic cups from the start of the phagocytosis till closure of phagocytic cups. Interestingly we observed that EhARPC1 was present just after the membrane fusion event but not when phagosome got separated from the membrane. The average time taken by the whole process was found to be 2s based on EhARPC1 enrichment.

**Fig 5:**
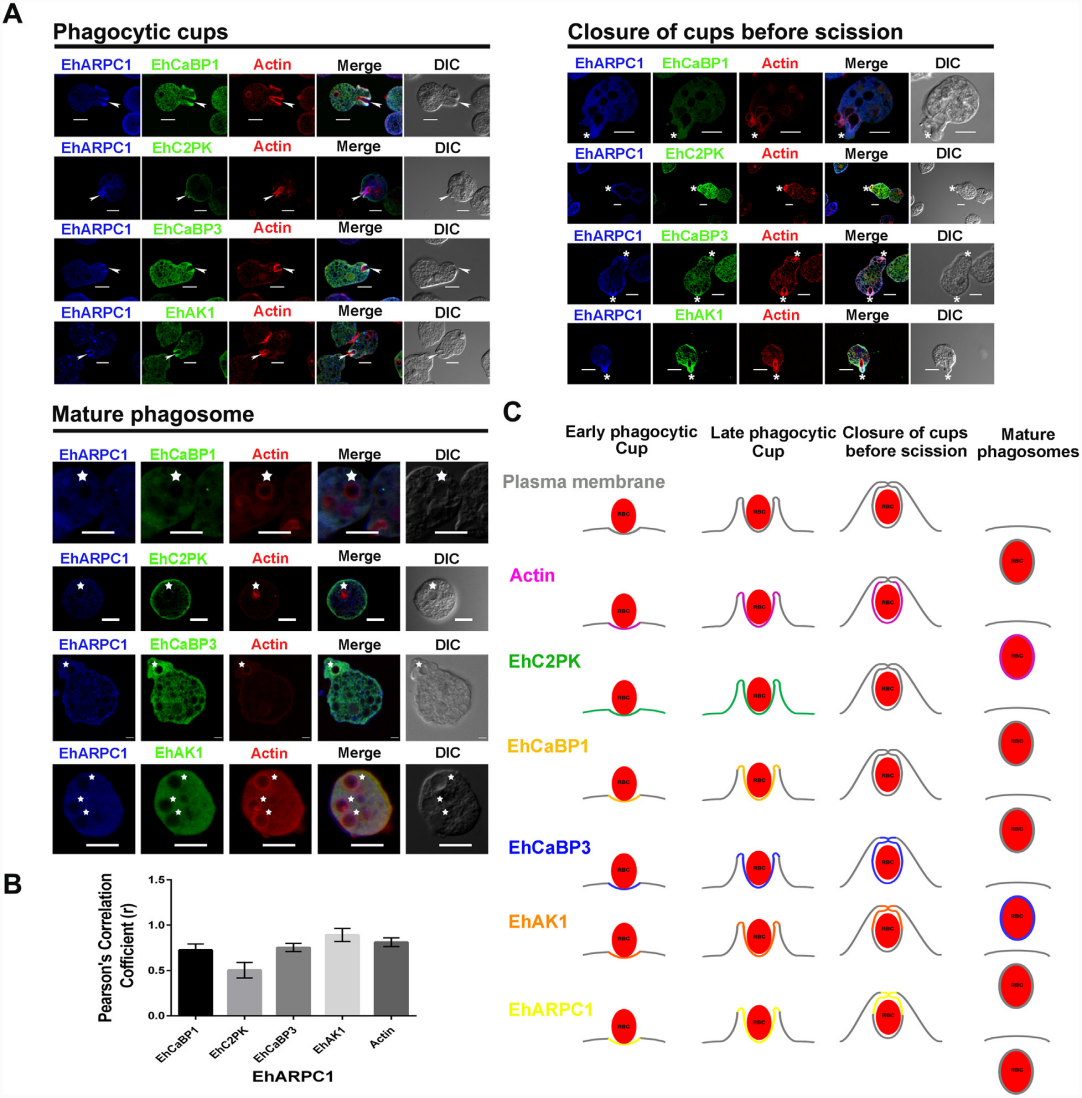
Pairwise colocalization of EhARPC1 with EhC2PK, EhCaBP1, EhCaBP3 and EhAK1 during phagocytosis of RBC. **(A)** *E*. *histolytica* cells were incubated with RBC for 5 min at 37°C. The cells were then fixed and immunostained with anti-EhARPC1 antibody followed by Pacific blue-410. F-actin was stained with TRITC phalloidin and other indicated proteins were immunostained with respective antibodies, followed by Alexa 488-labelled secondary antibody. Arrowhead indicate phagocytic cups, asterisk mark just closed cups before scission and star denotes phagosomes. Scale Bar represents 10 μm. **(B)** Colocalization analysis from 10 cells was done by using Olympus Fluoview FV1000 software. The Pearson’s coefficient (r) values of EhARPC1 with EhAK1, EhCaBP1, EhCaBP3 and EhC2PK from phagocytic cups are indicated. **(C)** Schematic representation of the different stages of phagocytosis and the localization of molecules (described in A) during these stages is summarized.

In order to understand the role of EhARPC1 in the context of some of the other molecules that have been identified as part of the phagocytosis pathway in *E*. *histolytica* (EhCaBP1, EhC2PK, EhCaBP3, EhAK1), pairwise staining was carried out and extent of co-localisation during phagocytosis was quantified using PCC (Fig 5A and 5B). We found all five molecules (actin, EhCaBP1, EhCaBP3, EhC2PK and EhAK1) in phagocytic cups along with EhARPC1. However, both EhCaBP1 and EhC2PK were not present in cups just closed before scission from membrane, and EhCaBP3 was the only molecule present in mature phagosomes. Therefore, it appears that EhARPC1 behaves like EhAK1 in terms of its association with phagocytic machinery. Both of these molecules leave phagosomes before or immediately after scission takes place. Summary of all observations are shown schematically in Fig 5C. All these results suggest that EhAK1 and EhARPC1 may participate in a similar manner during phagocytosis.

### Down regulation of EhARPC1 decreases phagocytosis

**Fig 6:**
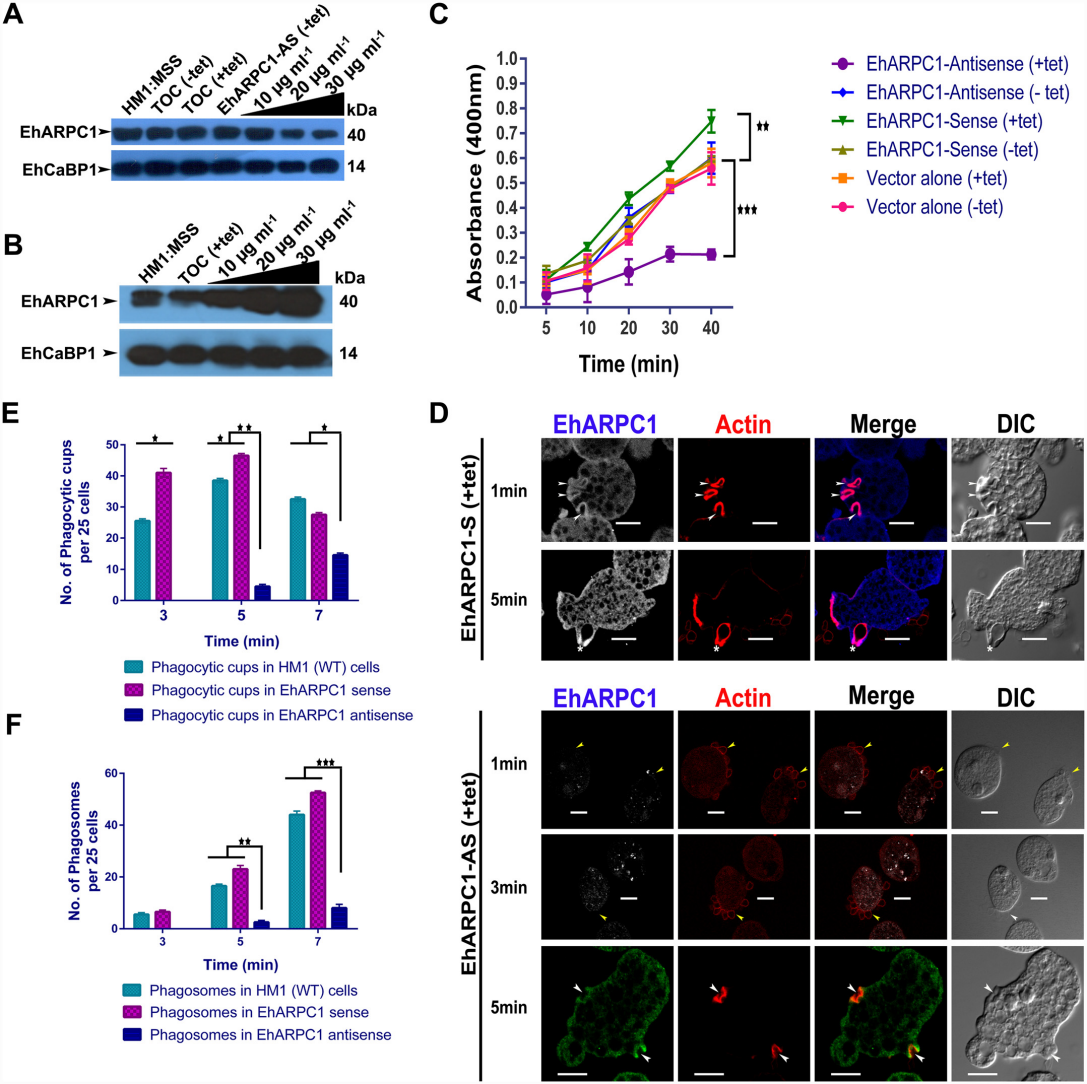
EhARPC1 is required for phagocytosis. **(A) and (B)** Immunoblot analysis of *E*. *histolytica* cells(HM-1:IMSS) expressing Tet-O-CAT (TOC) vector alone, or containing the cloned antisense EhARPC1 (AS) gene or sense EhARPC1 (S) in the presence and absence of tetracycline (30μg/ml). EhCaBP1 was used as an internal control. **(C)** Erythrocyte uptake assay was performed in over expressing (sense) and down regulated cell line (antisense) with vector alone as control. The assay was performed in the presence and the absence of tetracycline. The experiments were repeated independently three times. One-way ANOVA test was used for statistical comparisons. **(D)** Cells overexpressing either sense or antisense constructs of EhARPC1 were incubated with erythrocytes for the indicated times at 37°C. Cells were then fixed and stained for EhARPC1 (Alexa-488) and actin with TRITC-Phalloidin. Phagocytic cups are marked by arrowhead, star marks the just closed cup before scission and yellow arrowheads show attached RBCs at the site of phagocytosis **(E) and (F)** Quantitative analysis of phagocytic cups and phagosomes in over expressing (EhARPC1 sense), down regulating (EhARPC1 antisense) and Wild type HM1: MSS cell-lines, was carried out by randomly selecting 25 cells (in triplicates) and counting the number of phagocytic cups and phagosomes present in these cells. One-way ANOVA test was used for statistical comparisons. “One black star” p-value≤0.05, “Two black star” p-value≤0.005, “Three black star” p-value≤0.0005.

We further demonstrated the involvement of EhARPC1 in cytoskeleton dynamics by determining the effect of EhARPC1 down regulation on the rate of phagocytic cup formation, amoebic motility and proliferation. Down regulation of EhARPC1 expression was achieved by over expressing the gene in antisense orientation in a tetracycline-inducible manner [37–39]. The results are shown in Fig 6A. The level of down regulation achieved was 50% in the presence of 30 μg/ml tetracycline. On the other hand, EhARPC1 protein increased by 60% in cells expressing the gene in sense orientation (Fig 6B).

Phagocytosis of RBCs was measured in these cells using a colorimetric assay (Fig 6C). All comparisons were made against cells carrying either the vector alone, or with the gene construct in the absence of tetracycline. When EhARPC1 was over expressed in the sense cell line there was an increase in RBC uptake by 30% in 10 min. However, it was reduced by 70% in the antisense cell line in presence of tetracycline. We stained these cells with phalloidin and with antibodies against EhARPC1 and representative pictures are shown in Fig 6D and images depicting population of downregulated cells is shown in S3A Fig. In antisense expressing cells phagocytic cups were visible only after 5 min of incubation with RBC. We rarely observed phagosomes in these cells. In comparison, many phagocytic cups were visible in cells over expressing EhARPC1 by 1 min. We carried out quantitative analysis of the images by observing 25 cells (in triplicates) and enumerated number of phagocytic cups and phagosomes in these cells. By 5 min, cups and phagosomes were found to be only 11% and 7% of the control cells in antisense cells respectively. The results clearly showed that compared with wild type cells the rates of both cup and phagosome formation were significantly reduced in cells expressing antisense EhARPC1. On the other hand, cups and phagosomes increased by 60% and 40% respectively in cells over expressing EhARPC1 by 5 min (Fig 6E and 6F). Similar results were obtained when CHO cells were used for phagocytosis assay. Phagocytic cup formation was reduced significantly in the antisense cell line (S3B Fig). Further, cell motility and proliferation were also reduced in cells where EhARPC1 expression was down regulated by antisense RNA as compared to TOC vector alone in presence of tetracycline (S4 and S5 movies respectively). The level of reduction observed in antisense cells in case of proliferation was 40% (S3C Fig). From this data we can conclude that EhARPC1 is required for a number of process including phagocytosis in *E*. *histolytica*.

### EhAK1 recruits EhARPC1 at the site of phagocytosis

**Fig 7:**
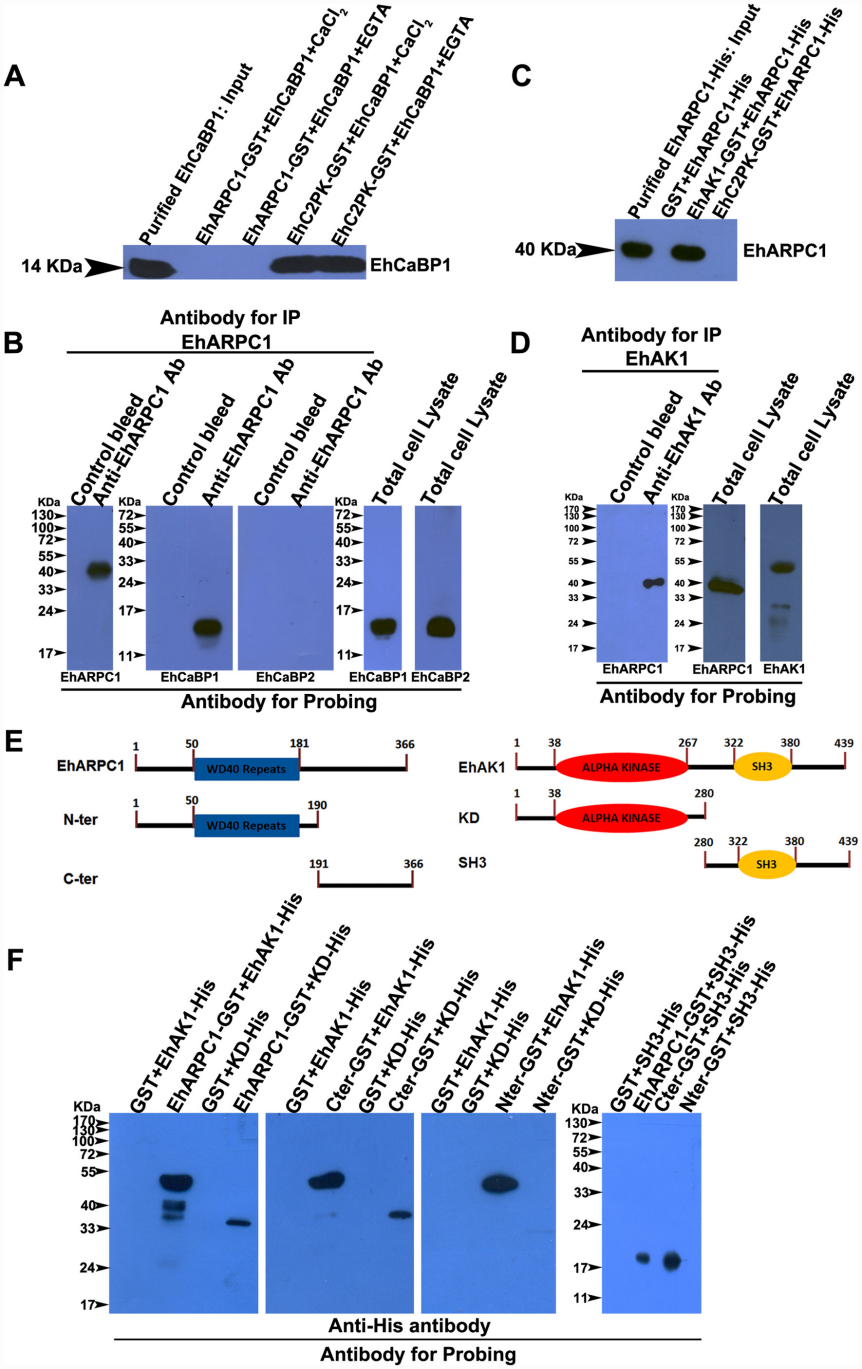
Binding of EhARPC1 with EhAK1. **(A)** Co-precipitation of EhCaBP1 with GST-tagged protein (GST-EhARPC1 or GST-EhC2PK) was tested using Glutathione Sepharose beads. Beads were first loaded with recombinant GST-tagged indicated proteins and then incubated in presence of recombinant EhCaBP1. Beads were washed, and eluted proteins were detected using anti-EhCaBP1 antibody in western blots. **(B)** Co-immunoprecipitation of EhCaBP1 from whole amoebic lysate was done using agarose conjugated with either anti-EhARPC1 antibody or pre-immune serum (PB). The beads were washed and the eluted proteins were probed by western blot analysis using indicated antibodies. **(C)** Co-precipitation of his-tagged EhARPC1 with GST-tagged EhC2PK and GST-tagged EhAK1 was carried out in similar way as mentioned in (b), proteins were detected with anti-his antibody. EhAK1 was able to precipitate his-tagged EhARPC1 whereas neither GST EhC2PK nor GST alone was able to precipitate his-tagged EhARPC1. **(D)** Immunoprecipitation of EhARPC1 from whole-cell lysate of *E*. *histolytica* using CNBr-conjugated anti-EhAK1 antibody. The total input lysate was used for the presence of EhARPC1 and EhAK1 using respective antibodies. **(E)** Schematic diagram showing the organization of EhAK1 (KD and SH3) and EhARPC1 (N-ter and C-ter) constructs. **(F)** Co-precipitation of his-tagged EhAK1, KD and SH3 with GST-EhARPC1, N-ter and C-ter was carried out in a similar way as mentioned above in (b), only EhARPC1 and N-ter was able to pull SH3 domains. All SDS-PAGE was carried out using 10%-12%polyacrylamide gels unless otherwise mentioned.

EhARPC1 was identified as EhCaBP1-binding protein in an affinity screen, as previously mentioned. In order to validate binding of EhARPC1 to EhCaBP1 directly we incubated GST-tagged EhARPC1 with EhCaBP1 in the presence and absence of Ca^2+^. Glutathione-Sepharose was used to pull down the complex and the presence of EhCaBP1 was determined by using a specific antibody. The result is shown in Fig 7A. No EhCaBP1 was found in the pull down material either in the presence or absence of Ca^2+^. As a positive control we used GST-tagged EhC2PK which directly binds EhCaBP1 [15], and could pull down EhCaBP1 both in the presence and absence of Ca^2+^ as expected. However, when we immunoprecipitated the complex from *E*. *histolytica* cell lysate using anti EhARPC1 antibody, we clearly observed EhCaBP1 in the pull down (Fig 7B), indicating indirect interaction between the two proteins.

We therefore tested if other EhCaBP1-binding proteins [30, 31] could act as a bridge between EhCaBP1 and EhARPC1. Recombinant EhAK1 and EhC2PK were used to test this in an *in vitro* pull down experiment. We incubated GST-tagged EhAK1 with full length His-tagged EhARPC1 and the pull down material was analysed by western blotting using anti-his antibody. A band corresponding to EhARPC1 was clearly seen in the pull-down (Fig 7C). However, no pull-down of EhARPC1 was observed using GST-tagged EhC2PK in a similar experiment (Fig 7C). The interaction of EhAK1 with EhARPC1 was further demonstrated by immunoprecipitation using a specific antibody and whole cell lysate. EhAK1 antibody indeed precipitated EhARPC1 from total lysate (Fig 7D). The results suggest a direct interaction of EhARPC1 with EhAK1, and indirectly with EhCaBP1.

We further investigated the binding between EhARPC1 and EhAK1 in order to understand the nature of interaction between these two molecules. For this, His-tagged fragments of EhAK1 containing either the kinase or the SH3 domain were generated as shown in Fig 7E. Although EhARPC1 is not known to be a multidomain protein, the molecule was divided into two parts, N terminal (Nter, containing WD 40 repeats) and C-terminal (Cter). Each fragment was fused to GST tag as outlined in Fig 7E. These fragments were used in the *in vitro* binding assay. GST-tagged full length EhARPC1, Nter and C-ter showed strong interaction with full length EhAK1 and weak binding with KD. The Nter fragment was able to bind to SH3 domain of EhAK1 as shown in Fig 7F. Since SH3 domains [40] and WD40 repeats [41] are known to be involved in protein-protein interaction, it is likely that EhARPC1 is recruited through the SH3 domain of EhAK1 through Nter.

**Fig 8:**
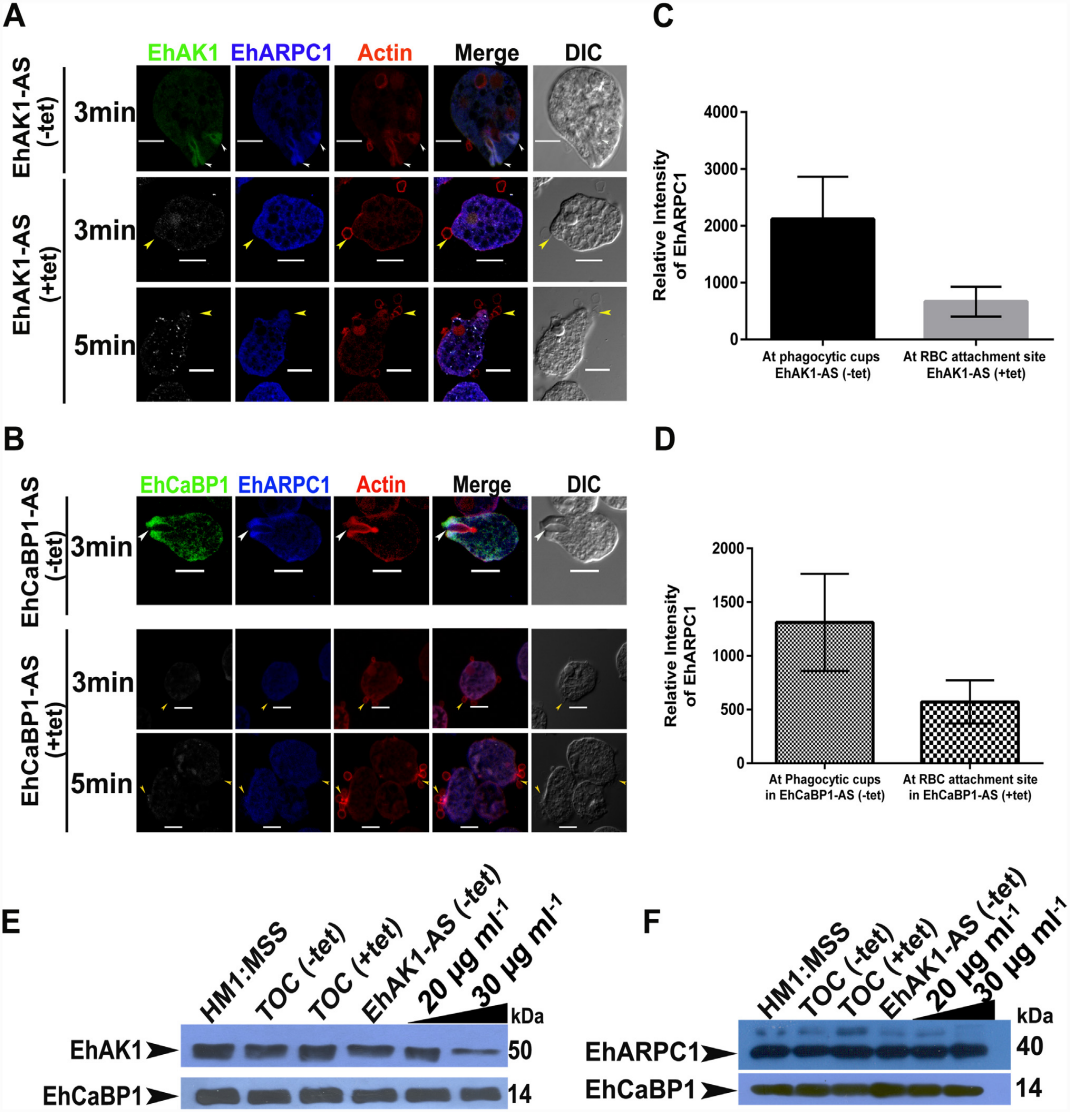
EhARPC1 is recruited at the site of phagocytosis via EhAK1. **(A)** and **(B)** Amoebic cells containing indicated constructs (EhAK1 or EhCaBP1) were grown for 48 h in the presence of 30μg/ml tetracycline (tet) and incubated with RBC for indicated times at 37°C. The cells were then fixed and immunostained with specific antibodies as indicated, and double stained with Alexa 488 (EhAK1/ EhCaBP1) or Pacific blue-410 (EhARPC1) labelled secondary antibodies. F-actin was stained with TRITC-phalloidin. Arrowheads indicate phagocytic cups formed in respective cell lines in absence of tetracycline and yellow color arrowheads indicate attached RBC in respective cell lines in presence of tetracycline. Alexa 488 staining of EhAK1/ EhCaBP1 in presence of tetracycline is pseudo-colored to gray. Scale Bar represents 10 μm. **(C) and (D)** Quantitative analysis of fluorescent signals of immunostained images of **(A)** is shown as a graph where N = 25 cells. One-way ANOVA test was used for statistical comparisons. **(E) and (F)** Western blot analysis of amoebic cells expressing indicated recombinant constructs showing the level of EhAK1 and EhARPC1 in tet-inducible vector alone, antisense EhAK1 (AS) or sense EhAK1(S) in the presence and the absence of tetracycline (30μg/ml). EhCaBP1 was used as an internal control. TOC is tet-o-CAT vector. “Two black star” p-value≤0.005.

We then studied if EhCaBP1 and its interacting partner EhAK1 may be involved in recruitment of EhARPC1 to the phagocytic cups. In order to demonstrate this, we visualized the sub-cellular localization of EhARPC1 in RBC phagocytosing cells carrying antisense constructs of either EhAK1 or EhCaBP1 in the presence and absence of tetracycline (Fig 8A and 8B). The results displayed that EhARPC1 was not enriched at the site of RBC attachment in these cell lines grown in presence of tetracycline upto 5 min of incubation with RBC (Fig 8A and 8B). Quantitative analysis indicated 60% reduction in the level of EhARPC1 signal at RBC attachment sites in EhAK1 antisense cells in presence of tetracycline as compared to level of EhARPC1 signal at the phagocytic cup in EhAK1 antisense cells in absence of tetracycline (Fig 8C). Similar results were obtained when EhCaBP1 levels were down regulated in antisense cell line in presence and absence of tetracycline (Fig 8D). In order to rule out any down regulation of EhARPC1 protein expression in EhAK1 antisense cells, we investigated the levels of EhARPC1 in EhAK1 antisense cell lines in presence of different concentrations of tetracycline. While there was a decrease in the level of EhAK1 and EhCaBP1 on increasing tetracycline concentration (Fig 8E and S4A Fig respectively), we did not observe any change in the level of EhARPC1 in these cells (Fig 8F and S4A Fig). Image depicting the population of downregulated EhAK1 and EhARPC1 cells is shown in S4B Fig. These results suggest that EhARPC1 recruitment at the phagocytic cups requires EhAK1 and on decreasing concentration of these molecules time taken to phagocytose RBC increase significantly.

### EhAK1 phosphorylates EhARPC1

**Fig 9:**
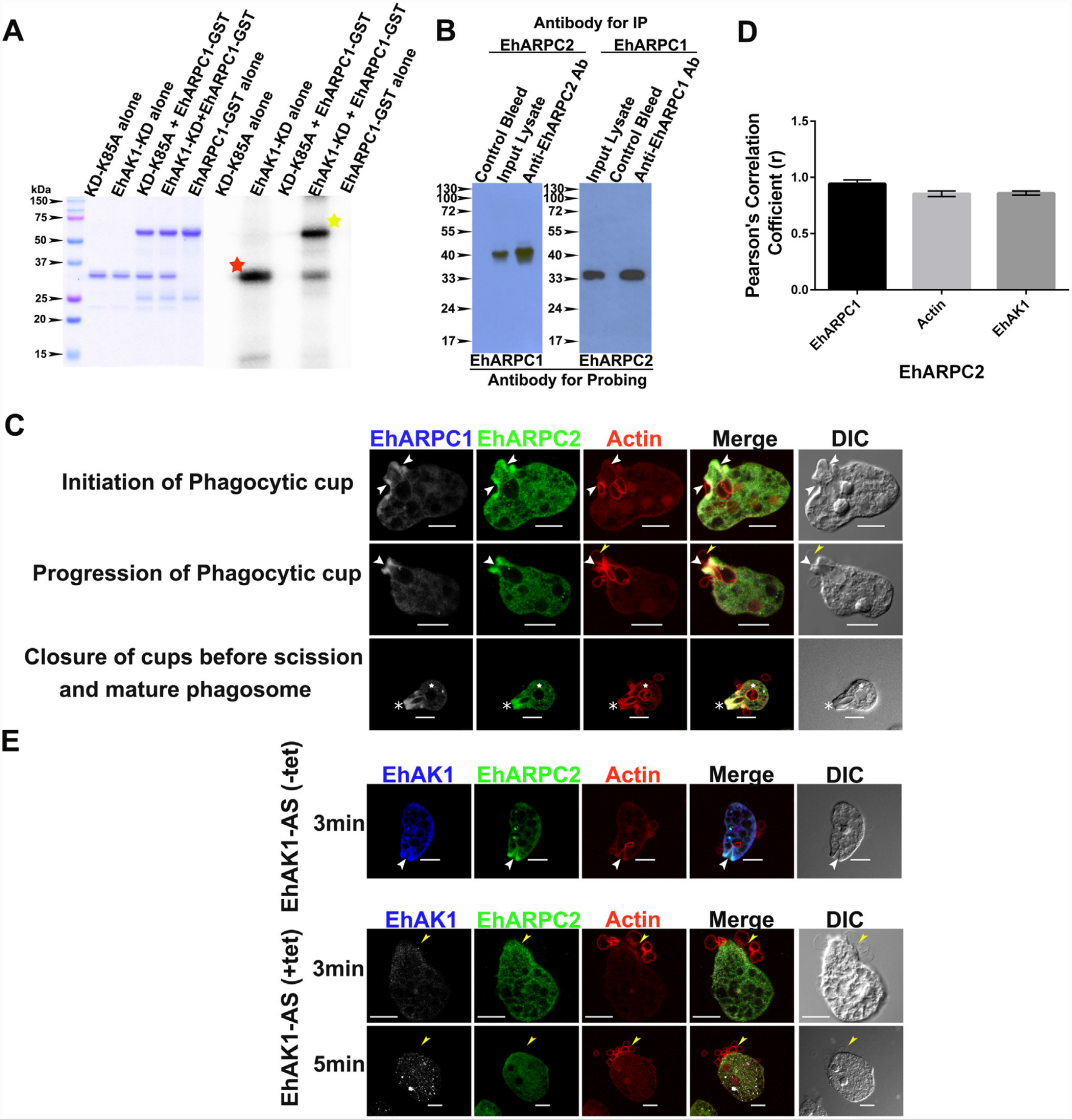
Recruitment of Arp2/3 complex at the site of phagocytosis. **(A)** Purified recombinant Kinase domain (KD) of EhAK1 or K85A (1μg) was incubated in the presence of γ-32P-ATP, MgCl^2^ and substrate EhARPC1-GST ((2μg) at 30°C for 1 h. KD showed phosphorylation of EhARPC1-GSTwhereas K85A mutant of EhAK1 exhibits no autophosphorylation and substrate phosphorylation activities. The products were analysed on SDS-PAGE and visualized in a phosphorimager. Red color star marks autophosphorylation band of EhAK1-KD and yellow color star marks substrate phosphorylation band. **(B)** Total cell- lysate of *E*. *histolytica* was passed through agarose conjugated with either anti-EhARPC1/ purified anti–EhARPC2 antibody or pre-immune serum. Co-Immunoprecipitation of EhARPC1 and EhARPC2 was checked using respective antibodies. **(C)** Imaging of EhARPC2 with respect to EhARPC1 and Actin was done during erythrophagocytosis where *E*. *histolytica* cells were incubated with RBCs for indicated times at 37°C. The cells were then fixed and immunostained with anti-EhARPC1 and anti EhARPC2 antibody followed by Pacific blue-410 and Alexa 488-labelled secondary antibodies respectively. F-actin was stained with TRITC phalloidin. Images with Pacific blue-410 labelled anti-EhARPC1 antibody were given pseudo-color to gray for efficient visualization. Arrowhead indicate phagocytic cups, asterisk closed cups before scission, star marks phagosome and yellow arrowheads indicate RBC to be phagocytosed. Scale Bar represents 10 μm. **(D)** Colocalization analysis and PCC (r) from 25 cells was done by using Olympus Fluoview FV1000 software. The values obtained by a pairwise analysis of EhARPC2 with EhARPC1, actin and EhAK1 from phagocytic cups are indicated. **(E)** Immunostaining was performed for amoebic cells containing EhAK1 antisense construct grown in presence or absence of 30μg/ml tet and were incubated with RBCs for 5min at 37°C. The cells were then fixed and immunostained with anti-EhAK1 and anti-EhARPC2 antibody as indicated and double stained with Pacific blue-410 and Alexa 488-labelled secondary antibodies respectively. F-actin was stained with TRITC-phalloidin. Images with Pacific blue-410 labelled anti-EhAK1 (in presence of tetracycline) is pseudo-color to gray for efficient visualization. Yellow color arrowheads show the site of RBC attachment. Scale Bar represents 10 μm.

Many protein kinases bind their cognate substrates. Since binding between EhARPC1 and the KD of EhAK1 was observed, we investigated if EhARPC1 is one of the substrates of EhAK1. The results are shown in Fig 9A. When purified KD of EhAK1 was incubated with EhARPC1 in presence of phosphorylation buffer and γ-^32^P-ATP, bands corresponding to phosphorylated form of EhARPC1 and autophosphorylated kinase were observed. No radioactive band was visible in the reaction were kinase dead mutant (K85A-EhAK1) was used as the enzyme. This suggests that in addition to actin [31], EhARPC1 is one of the substrates of EhAK1. The role of this phosphorylation event is not clear and is currently being investigated.

### The Arp 2/3 subunit EhARPC2 is also recruited at the site of phagocytosis

It is clear from the results presented so far that amoebic Arp 2/3 subunit EhARPC1 is recruited to the phagocytic site through EhAK1. Since Arp 2/3 is a complex of seven proteins it is likely that the whole complex may be recruited through EhARPC1 subunit. To demonstrate this, we investigated if another subunit of the complex might also be similarly recruited. ARPC2 has been used in the past as a representative subunit of the Arp 2/3 complex for determining presence of the entire complex [7, 42]. Moreover, crystal structure of bovine Arp2/3 complex and cross linking studies has revealed that Arpc1 and Arpc2 subunits are present in close proximity in the complex [43–45]. Therefore, we chose EhARPC2, the 34KDa subunit of the EhArp2/3 complex for further study, sequence alignment and specificity of anti-EhARPC2 antibody S5A and S5B Fig respectively. We first investigated possible interaction of EhARPC1 and EhARPC2 using an *in vitro* pull down approach. EhARPC1 antibody was able to pull EhARPC2 from total amoebic lysate (Fig 9B). Moreover, anti EhARPC2 antibody was also able to pull down EhARPC1 suggesting that these two proteins are present in the complex and may be interacting with each other, as seen in other systems.

We carried out fluorescence imaging during RBC uptake to localize EhARPC2 in relation to EhARPC1 (Fig 9C). Like EhARPC1, EhARPC2 was also found in phagocytic cups, and in just closed phagosomes, but not in phagosomes after scission. Quantitative analysis of the images displayed co-localization of both molecules at phagocytic sites (Fig 9D). Further we did not observe significant enrichment of EhARPC2 in RBC attachment sites of trophozoites expressing antisense EhAK1. Most of the stain was found in the cytoplasm (Fig 9E). The accumulation of EhARPC2 at RBC attachment sites in trophozoites expressing antisense EhARPC1 was also investigated. No significant enrichment at the site was observed suggesting that EhARPC2 is not recruited independent of EhARPC1 (S5C Fig). The results suggest that EhARPC2 is also recruited to the phagocytic site through EhAK1 and that EhARPC1 and EhARPC2 interact with each other. Therefore, the Arp2/3 complex is likely to be recruited to phagocytic sites through EhAK1.

## Discussion

Phagocytosis is a multifactorial and multistep process that is initiated on attachment of a particle and completed after phagosomes are formed and separated from plasma membrane. Attachment of the particle leads to activation of downstream signaling cascade which ultimately causes the tethering of actin filaments to the plasma membrane and generation of force required for the pseudopod protrusion. Therefore, one of the major objectives of the phagocytic signaling system is to initiate actin dynamics. The mechanism of coupling of the signaling system with that of actin dynamics has been worked out in a few systems. It appears that one of the key steps that initiates actin filament mesh is recruitment of proteins that are involved in nucleation, polymerization, bundling, and branching of actin. A few systematic studies were carried out to identify proteins that may be involved in phagocytosis. One approach used sequence similarity based identification of *E*. *histolytica* genome encoded homologs of known actin dynamics proteins with the assumption that some of these may be involved in actin dynamics during phagocytosis [11]. In the second approach proteins present in phagosomes under different conditions were characterized [12–14]. Though there is always the possibility that molecules that are involved in the early phase of phagocytosis may not be there by the time phagosomes are isolated, it is likely that the majority of the proteins involved at different phases may still be present in the proteome. We have used a different approach for identification of relevant proteins, particularly since we are interested in deciphering the pathway mediated by EhCaBP1. Our approach involved identification of proteins that bind EhCaBP1 and EhAK1 [30, 31]. EhARPC1 turned out to be one of the common proteins observed in most of the analysis described above (as shown in S1 Table). Since EhARPC1 is part of the Arp 2/3 complex proteins that are thought to be key regulators of actin dynamics we selected this protein for further studies. Involvement of other actin dynamics modulating proteins cannot be ruled out at present. Transient participation by these proteins may not likely be reflected in the proteome composition. The involvement of other pathways for phagocytosis in *E*. *histolytica* is an open question.

Our laboratory has investigated the sequence of events that are initiated on attachment of a particle destined for phagocytosis in *E*. *histolytica* [15, 30–32]. Though RBCs were mainly used as phagocytic particle, the proposed pathway was also found to operate during phagocytosis of mammalian cells [46]. The pathway unraveled so far proposes that upon particle attachment EhC2PK accumulates at the site, followed by EhCaBP1 and EhAK1. EhCaBP3 is independently recruited to the phagocytic sites. EhCaBP1, EhCaBP3 and EhAK1 bind actin and manipulate actin polymerization and/or bundling [15, 30–32]. However, these proteins are unlikely to be involved in actin nucleation and formation of directed branched filaments. In this report we show that the EhARPC1 and EhARPC2 of *E*. *histolytica* Arp 2/3 complex, are recruited to the macromolecular complex at the phagocytosis initiation site, and that this recruitment is through EhAK1. The results presented here show that the proposed phagocytic pathway involving EhARPC1 is also involved in the uptake of other cells, such as mammalian cells. The mechanism presented here for recruitment of Arp 2/3 complex proteins in *E*. *histolytica* has not been seen in any other system so far.

EhARPC1, the p41 subunit of Arp 2/3 complex was first identified as EhCaBP1 binding protein through a proteomic screen [30]. However, results presented here clearly show that EhARPC1 binds EhAK1, and it interacts with EhCaBP1 through EhAK1. Moreover, our results suggest that N-terminal domain consisting of WD40 repeats of EhARPC1 binds mainly the SH3 domain of EhAK1. Since SH3 domains are known to be involved in protein-protein interaction and act as recruiters of various molecules at signaling sites it was not surprising to find this domain playing a possible role in EhARPC1 recruitment [40]. A number of evidences suggest that the interaction between EhAK1 and EhARPC1 is important for RBC uptake in these cells; EhARPC1 is not recruited to the phagocytic cups on down regulation of EhAK1 expression and similar pattern of distribution of EhARPC1 and EhAK1 during phagocytosis, that is, present in phagocytic cups and just closed phagosomes, but not in phagosomes after scission, unlike EhC2PK, EhCaBP1 and EhCaBP3. Similar observations were also made in *D*. *discoideum* using GFP-tagged Arp3 and p41Arc. These proteins were present just after closure of phagosomes, but not after phagosomes were separated [47]. This is consistent with the role of Arp 2/3 complex in generating the necessary force for extension of pseudopods. Once phagosomes are formed there is no need for proteins that are involved in actin branching and extension. On phagosome maturation, actin molecules are removed paving the way for vesicles to fuse with other compartments [48, 49].

We have also looked into the possibility that EhARPC1 recruitment at the cups is needed for progression of cups to become phagosomes. This was done by observing the effect of expression knockdown of EhARPC1 on amoebic phagocytosis. A significant reduction in phagocytic rate suggested that recruitment of EhARPC1 is necessary both for initiation (delay in cup formation), and completion of phagocytosis (delay in phagosome formation).

ARPC1 is an important subunit of the Arp 2/3 complex. In *S*. *cerevisiae* null mutants of all Arp 2/3 complex proteins except Arpc1 survive, suggesting that this is the only essential subunit of Arp 2/3 complex [50]. Similar studies in *A*. *thaliana* have also shown that T-DNA insertion mutants of Arp2, Arp3 and ARPC5 did not have major defects in development and were still viable, but ARPC1 was essential [51]. However, Arp2 was also found to be essential in *D*. *discoideum*, and *E*. *histolytica* homolog was able to complement *D*. *discoideum* protein suggesting that some of these proteins from *E*. *histolytica* may be functionally equivalent to that from other systems [52]. Our sequence analysis did reveal that EhARPC1 may have different properties compared to ARPC1 from other organisms unlike Arp2 subunit, which may be more conserved.

It is likely that Arp 2/3 complex is recruited to phagocytic sites through EhARPC1. In order to show if the whole complex is likely to be present and not just EhARPC1, we have used EhARPC2 or p34 subunit as a marker of Arp 2/3 complex. This subunit of Arp 2/3 complex has been used as a representative of Arp 2/3 complex in many studies [7, 42]. A number of observations, such as imaging, and pull down suggest that both EhARPC1 and EhARPC2 interact with each other and colocalize at the phagocytic cups. We have also shown that EhARPC2 is not recruited independent of EhARPC1. Down regulation of EhARPC1 expression abolished EhARPC2 accumulation at the site of phagocytosis. Therefore, it appears that EhAK1 recruits both EhARPC1 and EhARPC2 to the phagocytic site. Additional evidence in support of this comes from our previous study where we had observed that EhAK1 could pull down both EhARPC1 and EhARPC2 [31]. Our data show that during phagocytosis coupling of the signaling system to actin dynamics is likely to be mediated through recruitment of EhARPC1 and EhARPC2, components of Arp 2/3 complex through EhAK1. The role of ARPC1 (also known as Arc 40) in the recruitment of Arp 2/3 complex has also been shown in yeast [33, 50]. Here, the ARPC1/Arc40 protein binds the VCA domain of WASP activators and helps Arp 2/3 complex recruitment through an “Arm” region at the C-terminal of Arc40. However, in our study we find that EhARPC1 is likely to be recruited by binding with SH3 domain of EhAK1. We have also seen EhAK1 kinase domain binding EhARPC1. Since many protein kinase substrates are known to interact with respective kinases [53], it was speculated that EhARPC1 may be a substrate of EhAK1. This was verified experimentally and EhAK1 dependent phosphorylation of EhARPC1 was observed. In our previous study actin was shown to be a major substrate of EhAK1 based on use of total amoebic cell lysate [31]. In view of the results presented here it appears that though actin may be the major substrate, EhAK1 may also be phosphorylating other proteins, as minor substrates. The significance of EhARPC1 phosphorylation in relation to mechanism of phagocytosis is currently being explored. ARPC1 is known to be phosphorylated in mammalian systems by p-21 activated kinase. This phosphorylation is required for optimal cell motility upon stimulation with growth factors and may play a critical role for localization of this subunit with rest of the complex [54]. Phosphorylation of ARPC1 by an alpha kinase like kinase has not been observed before.

It is also possible that EhARPC1 has a functional role independent of being part of Arp 2/3 complex. This may likely explain the essential nature of this protein unlike other components of Arp 2/3 complex. Whether EhARPC1 also has other functions in *E*. *histolytica* is totally an open question. In conclusion a novel mechanism of recruitment of Arp 2/3 complex to the phagocytic machinery in *E*. *histolytica* is proposed. This suggested mechanism is distinctly different from all other mechanisms proposed so far. Since this parasite has a high phagocytic rate it may have evolved novel mechanisms to meet its requirement for rapid actin dynamics.

## Materials and Methods

### Ethics statement

Both mice and rabbits used for generation of antibodies were approved by the Institutional Animal Ethics Committee (IAEC), Jawaharlal Nehru University (IAEC Code No.: 18/2010). All animal experimentations were performed according to the National Regulatory Guidelines issued by CPSEA (Committee for the Purpose of Supervision of Experiments on Animals), Ministry of Environment and Forest, Govt. of India.

### Growth, maintenance and transfection of *E*. *histolytica*

*E*. *histolytica* strain HM-1: IMSS trophozoites and all transformed strains were maintained and grown in TY1-S-33 medium supplemented with 125 μl of 250 U ml^− 1^penicillin G (potassium salt from Sigma) and 0.25 mg ml^− 1^streptomycin per 100 ml of medium as described before [37]. The transformants containing tetracycline inducible system and GFP (a constitutive expression system) were grown in the presence of 10 μg ml^− 1^of hygromycin B or G418. The cells were first grown for 48 h (60–70% confluent) and then 30 μg ml^− 1^ tetracycline or 20 μg ml^− 1^ G418 was added to the medium for 36 h for induction.

*E*. *histolytica* was transfected by electroporation. Briefly trophozoites were collected from log phase cultures and washed with PBS followed by incomplete cytomix buffer (10 mM K_2_HPO_4_/KH_2_PO_4_ (pH 7.6), 120mMKCl, 0.15mM CaCl_2_, 25 mM HEPES (pH 7.4), 2 mM EGTA, 5 mM MgCl_2_). The washed cells were then re-suspended in 0.8 ml of complete cytomix buffer (incomplete cytomix containing 4 mM adenosine triphosphate, 10 mM glutathione) containing 200 μg of plasmid DNA and subjected to two consecutive pulses of 3,000 V cm^− 1^(1.2 kV) at 25 mF (Bio-Rad, electroporator). The transfectants were initially allowed to grow without any selection. Drug selection was initiated after 2 days of transfection in the presence of 10 μg ml^−1^G418 (for constitutive expression vectors) or hygromycin B (for tetracycline inducible vector)

#### Cloning of various constructs

The CAT gene of the shuttle vector pEhHYG-tet-O-CAT was excised using *Kpn*I and *Bam*HI and *EhARPC1*gene was inserted in its place in either the sense or the antisense orientation. The full-length gene was cloned in *Bam*H1 site in the case of GFP vector resulting in GFP tag on amino terminal of protein. Oligonucleotide sequences for making the above stated constructs are as follows: FSense-5’CGGGGTACCATGTCAGCTCCAAAGAGTTTCC3’RSense- 5’CGCGGATCCTTAAGCTTTCCAAATGGCTATATTACC3’ FAntisense- 5’ CGCGGATCCATGTCAGCTCCAAAGAGTTTCC3’RAntisense-5’CGGGGTACCTTAAGCTTTCCAAATGGCTATATTACC3’ ARP GFP F: 5’CAGGATCCATGTCAGCTCCAAAGAGTTTCC3’ ARP GFP R: 5’CGCGGATCCTTAAGCTTTCCAAATGGCTATATTACC3’

#### Immunofluorescence staining

*E*. *histolytica* trophozoites were centrifuged and re-suspended in incomplete TY1-33 medium. Further cells were transferred onto acetone-cleaned coverslips placed in a petri dish. The cells were allowed to adhere for 5 min at 37°C. The culture medium was discarded and the cells were fixed with 3.7% pre-warmed paraformaldehyde for 30 min. After fixation, the cells were permeablized with 0.1% Triton X-100/PBS for 3 min. The fixed cells were then washed with PBS and quenched for 30 min in PBS containing 50 mM NH_4_Cl. The coverslips were blocked with 1% BSA/PBS for 2h, followed by incubation with primary antibody at 37°C for 1.5h. The coverslips were washed with PBS followed by 1% BSA/PBS before incubation with secondary antibody for 30 min at 37°C. Antibody dilutions used were: anti-EhARPC1/anti-EhARPC2/anti-EhAK1 at 1:100, anti-EhCaBP1/anti-EhCaBP3/anti-EhC2PK at 1:200, anti-rabbit/mice Alexa 488, Alexa 556 and Pacific blue-410 (Molecular Probes) at 1:250, TRITC-Phalloidin at 1:250. The preparations were further washed with PBS and mounted on a glass slide using DABCO (1,4-diazbicyclo (2,2,2) octane (55) 2.5% in 80% glycerol). The edges of the coverslip were sealed with nail-paint to avoid drying. Confocal images were visualized using an Olympus Fluoview FV1000 laser scanning microscope.

#### Western blotting

For immunoblotting, samples were prepared in 2X SDS Polyacrylamide gel electrophoresis (PAGE) buffer followed by separation on 10–12% SDS–PAGE as required. The gel was then transferred on to a polyvinylidine fluoride (PVDF) membrane using semi dry transfer system. The antigens were detected with polyclonal antibodies raised in rabbit and mice at a dilution of 1:1000 followed by secondary anti-rabbit and anti-mice immunoglobulins conjugated to HRPO (1:10,000, Sigma). ECL reagents were used for visualization (Millipore). GST/His antibodies were used at a dilution of 1:3000 and was obtained from Sigma. The concentration of proteins in a sample was estimated by bicinchoninic acid assay using BSA as a standard.

#### Erythrophagocytosis assay

Equal number of RBCs and *Entamoeba* (10^5^) were harvested via centrifugation and were washed with PBS and incomplete TYI-33 medium respectively. Further RBC’s were incubated with 10^5^entamoeba for varying times as indicated at 37°C in 0.5 ml of culture medium. The amoebae and erythrocytes were centrifuged and cold distilled water was added to lyse the non-engulfed RBCs and re-centrifuged at 1,000 *g* for 2 min. This step was repeated twice, followed by re-suspension in 1 ml formic acid to lyse *Entamoebae* containing engulfed RBCs. The absorbance was measured at 400 nm with formic acid as blank.

#### Fluorescent labelling of Chinese Hamster Ovary (CHO) cells

CHO cells (obtained from Cell Repository-National Centre for Cell Science, Pune, India) were labelled by blue CMAC (7-amino-4-chloromethylcoumarin) dye (Life Technologies) following manufacturer’s protocol for adherent cells. Briefly 10^5^ cells were stained for 30min with 5μM pre-warmed cell tracker dye diluted in serum free medium. After staining CHO cells were washed thrice with fresh medium and approximately 4×10^5^ CHO cells were incubated with 2×10^5^ cells of amoeba expressing GFP-EhARPC1 for Time lapse imaging and at indicated time points for immunofluorescence. In time lapse imaging and immunostaining live blue color CHO cells were incubated with amoeba.

#### Fluorescent labelling of RBCs

RBCs were stained with CFDA (Carboxyfluorescein succinimidyl ester, Thermo fisher Scientific Cat no C1157). Cells (2X10^7^ cells/ml) were washed with PBS containing 0.1% BSA thrice followed by incubation in CFDA staining buffer (PBS containing 0.1% BSA and 10μM CFDA) for 10 min at 37°C with intermittent tapping. The reaction was stopped with pre chilled complete medium with 2% serum for 10 min on ice, followed by washing of RBC with *E*. *histolytica* incomplete media thrice.

#### GST-bead pull down assay

Purified GST-EhARPC1 was incubated with Glutathione beads (Amersham) for 1 h at 4°C in buffer comprising of 10mM Tris-Cl (pH 7.5), 0.1mM EDTA, 0.1% NP-40 (w/v), 2mM DTT, 100mM NaCl, 0.2mM PMSF. Then EhCaBP1 or his tagged proteins as indicated was added to the reaction and incubated for 2 h at 4°C. The beads were then washed with washing buffer comprising of 10mM Tris-Cl, 1% Glycerol, 1mM EDTA, 0.1% NP-40, 2mM DTT, 100mM NaCl, and 0.2mM PMSF thrice. Protein was eluted by adding 2X SDS–PAGE buffer followed by boiling for 5 min. The proteins were then analysed by western blotting. The same procedure was followed for other proteins with GST-tags.

#### Immunoprecipitation

Cell lysate for immunoprecipitation contained 10 mMTris-HCl, pH 7.5, 150 mMNaCl, 1 mM phenylmethylsulfonyl fluoride (PMSF), protease inhibitor cocktail, 2 mM β-ME and 1% Triton 100 and was prepared as described before^15^. It was centrifuged at 15,000 rpm to remove all cellular debris. Anti-EhAK1 antibody was conjugated to CNBr-activated Sepharose (1 g, Pharmacia) that was activated and processed as per the manufacturer’s protocol. In brief, 40% ammonium sulphate was used to collect crude immunoglobulins from the immunized serum. The obtained immunoglobulins were then dialysed in coupling buffer (bicarbonate buffer). Usually, 10 mg protein was added per gram of resin. The resin was mixed gently for 18 h at 4°C. After coupling the coupled resin was processed as per manufacturer’s protocol. The conjugated CNBr-Sepharose beads were incubated with *E*. *histolytica* lysate (500mg) for 4 h at 4°C.

The beads were then washed with wash buffer (10 mMTris-Cl (pH 7.5), 150 mMNaCl, 1 mM imidazole, 1 mM magnesium acetate, 2mM β-ME, 0.1% Triton X 100 and protease inhibitor cocktail) thrice. Beads were washed with 0.06 mMTris-Cl (pH 6.8) and 100 mMNaCl and finally with 0.06 mM Tris-Cl (pH 6.8). The pellet was suspended in 2XSDS polyacrylamide gel electrophoresis (PAGE) buffer and boiled for 5 min followed by centrifugation for 5 min.

The proteins were then analysed by western blotting. For immunoprecipitation of EhARPC1/EhARPC2, 10μl of anti-EhARPC1 or anti-EhARPC2 antibody at 1:1000 dilution was incubated with pre-cleared amoebic lysate and then allowed to bind with protein A or protein G Sepharose beads (Amersham) for 2 h at 4°C. Thereafter the same protocol was followed as described above.

#### Kinase assay

Phosphorylation by EhAK1 (Substrate phosphorylation) was analysed as the amount of radioactivity incorporated (γ-32P-ATP) into the purified recombinant EhARPC1-GST The standard kinase reaction mixture (40 μl final volume) contained 0.5 mM MgCl_2_, 30 mM HEPES (pH 7.5), protease inhibitor, phosphatase inhibitor cocktail, EhAK1 (1 μg) and EhARPC1 (2 μg). Reactions were initiated by the addition of (γ-32P-ATP) (6000 Ci/mmol) to a final concentration of 2.5 μM and incubated at 30°C for 1 h and was stopped by adding SDS sample buffer containing 50 mM EDTA followed by boiling. The samples were than resolved on SDS-PAGE. Radioactive bands were detected by a Phosphor Imager (GE Healthcare).

#### Time-lapse imaging

The amoebic cells expressing GFP-EhARPC1 were plated onto a 35 mm glass bottom dish. The amoeba was allowed to settle down and get adhered to the plate. The temperature of dish was maintained at 37°C by keeping it on a temperature controller platform. Spinning Disk confocal microscope was used for fluorescent time-lapse imaging (Nikon A1R, Optics- Plan Apo VC606 oil DIC N2, Camera- Nikon A1, NA-1.4, RI-1.515) of a moving and phagocytosing amoeba was performed. The images were captured at 10ms interval. The raw images were processed using NIS element 3.20analysis software.

#### Statistical analysis

Statistical comparisons were made using a one-way ANOVAtest. Experimental values were reported as the means ±s.e. Differences in mean values were considered significant at “one black star” p-value≤0.05, “two black star” p-value≤0.005, “three black star” p-value≤0.0005. All calculations of statistical significance were made using the Sigma plot software and Graph pad prism. Pearson’s Correlation Coefficient was obtained using Olympus Fluoview FV1000 software or JACoP for co-localization a plugin of Image J software available freely on the web (http://rsb.info.nih.gov/ij/).

## Supporting Information

S1 Fig Schematic presentation of domain organization of ARPC1 (subunit 1) of Arp2/3 complex. **(A)** Sequence alignment of *E*. *histolytica* ARPC1 with Arp2/3 complex subunit 1 from *Saccharomyces cerevisiae*, *Dictyostelium discoideum*, *Homo sapiens*, *Bostarus*, *Schizosachromyces pombe*, *Arabidopsis thaliana*. **(B)** Domain organization of ARPC1 from different organisms is indicated. WD40 repeats are marked by a box, highlighting the difference in the length of the repeats in different organisms. **(C)** Sequence alignment of *E*. *histolytica* and *S*. *cerevisiae* ARPC1. It is depicting the absence of "Arm” region required for binding of WASP *S*. *cerevisiae* is (underlined by blue line) is not conserved in EhARPC1.
(TIF)

S2 Fig EhARPC1-GFP shows similar cellular localization as endogenous EhARPC1. **(A)** Western blot analysis was performed for checking the specificity of Anti-EhARPC1 antibody raised against recombinant protein. Entamoeba lysate (75μg) was probed with Anti-EhARPC1 (1:1, 000). Pre-bleed was taken as control. Expected size of EhARPC1 is 41 KDa **(B)** Quantitative analysis of colocalisation of EhARPC1-GFP with endogenous EhARPC1 or Actin was carried out using Pearson’s correlation coefficient (r) using ten stained images. **(C)** Intensity profiles represent the fluorescence intensity of EhARPC1, EhAK1 and EhTMKB1-9 in cytoplasm and membrane. Results were calculated from ten randomly selected cells using Image J software. Snapshot of ROI (a line across the cell) selected for quantification in a single cell is shown in the box. **(D)** *E*. *histolytica* cells were incubated with CFSE labelled green colored RBC’s for different time points and then cells were fixed and stained for EhARPC1 antibody followed by Alexa 555. **(E)** *E*. *histolytica* cells either expressing GFP alone (top panel) or EhARPC1-GFP (lower panel) were incubated with RBC for 5 min at 37°C. The cells were then fixed and immunostained with anti-EhARPC1 antibody followed by Pacific blue-410. F-actin was stained with TRITC phalloidin and EhARPC1-GFP was immunostained with anti-GFP antibody followed by Alexa 488-labelled secondary antibody. Arrowhead indicate phagocytic cups, asterisk mark just closed cups, star denotes phagosome and yellow color arrowhead mark attached RBC. Scale Bar represents 10 μm.
(TIF)

S3 Fig Downregulation of EhARPC1 delays phagocytosis of RBC and mammalian cells. **(A)** *E*. *histolytica* trophozoites expressing anti sense EhARPC1 RNA were incubated with RBC for indicated time interval (3, 5 and 7 min) at 37°C. The cells were then fixed and immunostained with EhARPC1 antibody followed by Alexa 488. Actin was stained with TRITC-phalloidin. Green color is pseudo-colored to gray for efficiently showing the low fluorescent signals from EhARPC1-AS cell line. **(B)** Cells overexpressing either sense or antisense constructs of EhARPC1 were incubated with cell tracker blue dye-labelled live CHO cells for the indicated times at 37°C. Cells were then fixed and stained for EhARPC1 followed by Alexa-488conjugated secondary antibody. Phagocytic cups are marked by arrowhead, star marks just closed cup and yellow arrowhead show attached CHO cells at the site of phagocytosis. **(C)** Proliferation of *E*. *histolytica* trophozoites carrying different constructs was studied. All cells were grown in presence of 10 μg/ml hygromycin and tetracycline was added to the medium at 30 μg/ml at 0 h. Cells were grown in 5 ml culture tubes in triplicate for all the experiments and counting was carried out using a haemocytometer, after chilling the tube for 5 min. One-way ANOVA test was used for statistical comparisons. “Two black star”p-value≤0.005.
(TIF)

S4 Fig Levels of EhCaBP1 in its antisense RNA expressing cell lines. **(A)** Western blot analysis of amoebic cells expressing antisense EhCaBP1 RNA showing the level of EhCaBP1 and EhARPC1 in tet-inducible vector alone, antisense EhCaBP1 (AS in the presence and the absence of tetracycline (30μg/ml). EhARPC1 was used as an internal control. TOC is tet-o-CAT vector. **(B)** *E*. *histolytica* trophozoites expressing either anti sense EhCaBP1 or EhAK1 RNA were incubated with RBC for 5min time interval at 37°C. The cells were then fixed and immunostained with EhCaBP1 or EhAK1 antibody followed by Alexa 488. Actin was stained with TRITC-phalloidin. Green color is pseudo-colored to gray for efficiently showing the low fluorescent signals from EhCaBP1-AS and EhAK1-AS cell lines.
(TIF)

S5 Fig EhARPC1 recruits EhARPC2 at the site of phagocytosis. **(A)** Sequence alignment of *E*. *histolytica* ARPC2 with Arp2/3 complex subunit 2 from *Saccharomyces cerevisiae*, *Dictyostelium discoideum*, *Homo sapiens*, *Bostarus*, *Schizosachromyces pombe*, *Arabidopsis thaliana*. **(B)** Western blot analysis was performed for checking the specificity of Anti-EhARPC2 antibody raised against recombinant protein. Entamoeba lysate (100μg) was probed with Anti-EhARPC2 (1:1, 000). Pre-bleed was taken as control. Expected size of EhARPC2 is 34 KDa. **(C)** For immunostaining, amoebic cells containing EhARPC1 antisense construct in which cells were grown for 48 h in the presence of 30μg/ml tet were incubated with RBCs for indicated times at 37°C. The cells were then fixed and immunostained with anti-EhARPC1 and anti-EhARPC2 antibody as indicated and double stained with Pacific blue-410 and Alexa 488-labelled secondary antibodies respectively. F-actin was stained with TRITC-phalloidin. Yellow arrowhead shows the site of RBC attachment. Scale Bar represents 10 μm.
(TIF)

S1 Movie Live cell imaging of GFP-EhARPC1 during pseudopod formation. The movie represents pseudopod formation in GFP-EhARPC1 expressing trophozoites. The enrichment of EhARPC1 is visualized at the leading edge of amoebae. Bar represents 10 μm.
(AVI)

S2 Movie Live cell imaging of GFP-EhARPC1 during erythrophagocytosis. The movie represents the erythrophagocytosis process and the enrichment of EhARPC1 at the phagocytic cups during this process. Bar represents 10 μm.
(AVI)

S3 Movie Live cell imaging of GFP-EhARPC1 during uptake of mammalian cells. The movie represents the involvement of EhARPC1 during the uptake of CHO cells. Bar represents 10 μm.
(AVI)

S4 Movie Live cell imaging of amoebic trophozoites expressing antisense EhARPC1 RNA. The movie represents the effect on motility of amoeba, expressing EhARPC1 antisense RNA. Bar represents 10 μm.
(AVI)

S5 Movie Live cell imaging of amoebic trophozoites expressing Tet-O-CAT vector alone. The movie represents the effect on motility of amoeba, expressing Tet-O-CAT vector alone. Bar represents 10 μm.
(AVI)

S1 Table Actin binding proteins involved in cytoskeleton dynamics (and phagocytosis) in *E*. *histolytica*. (DOCX)

S2 Table Table summarizing the percentage identity of *E*. *histolytica* proteins involved in actin dynamics with respect to proteins from other organism. (DOCX)

S3 Table List of antibodies used in the study. (DOCX)

S1 References References cited in Supporting Information files. (DOCX)
