# Supplementary figures and images for: The Entamoeba histolytica, Arp2/3 Complex Is Recruited to Phagocytic Cups through an Atypical Kinase EhAK1
Source: PLoS Pathog. 2015 Dec 8;11(12):e1005310. doi: 10.1371/journal.ppat.1005310 (PMC4672914; doi:10.1371/journal.ppat.1005310)

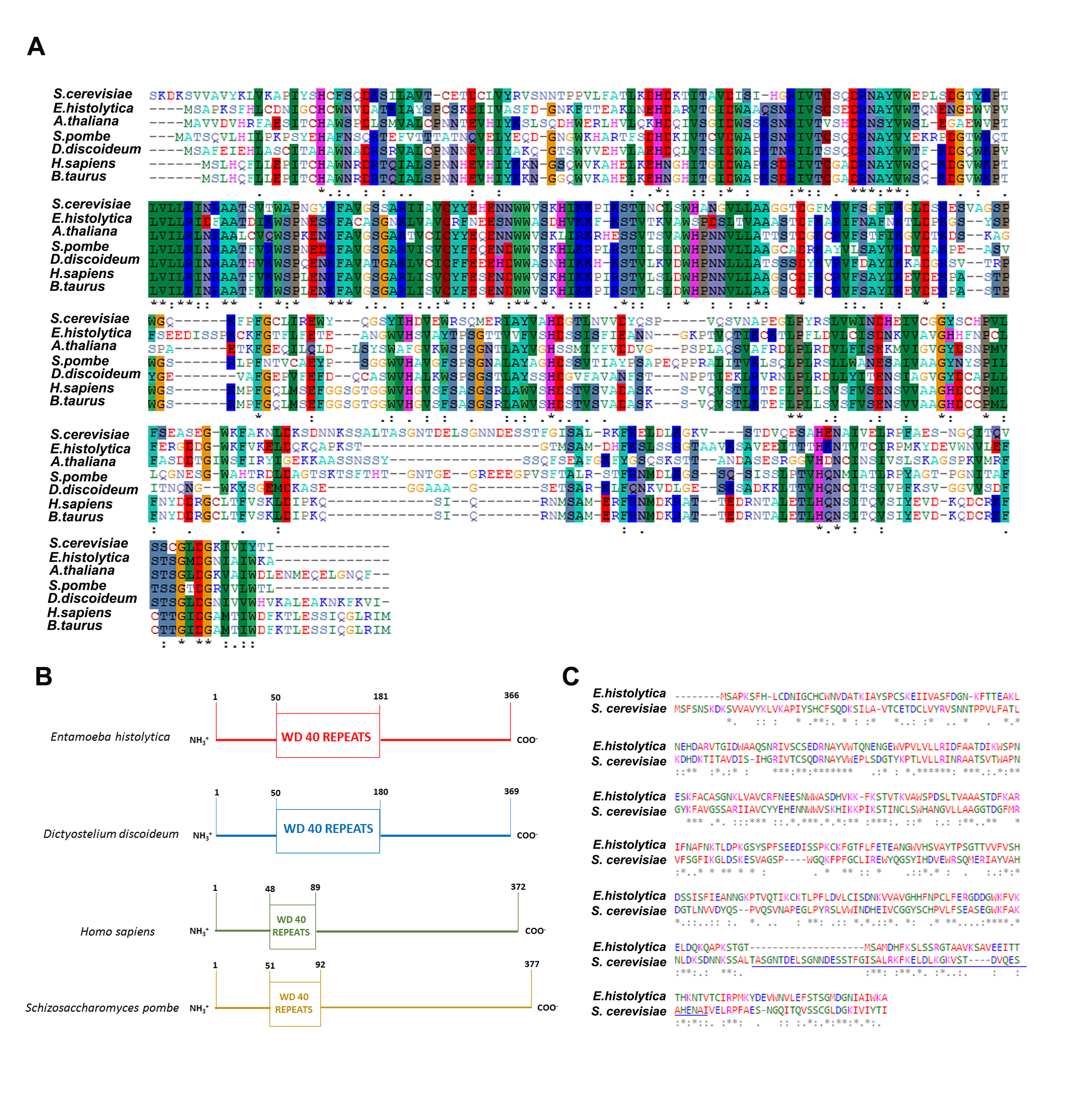

Supplement: S1 Fig — (A) Sequence alignment of E. histolytica ARPC1 with Arp2/3 complex subunit 1 from Saccharomyces cerevisiae, Dictyostelium discoideum, Homo sapiens, Bostarus, Schizosachromyces pombe, Arabidopsis thaliana. (B) Domain organization of ARPC1 from different organisms is indicated. WD40 repeats are marked by a box, highlighting the difference in the length of the repeats in different organisms. (C) Sequence alignment of E. histolytica and S. cerevisiae ARPC1. It is depicting the absence of "Arm” region required for binding of WASP S. cerevisiae is (underlined by blue line) is not conserved in EhARPC1. (TIF) [file ppat.1005310.s001.tif]

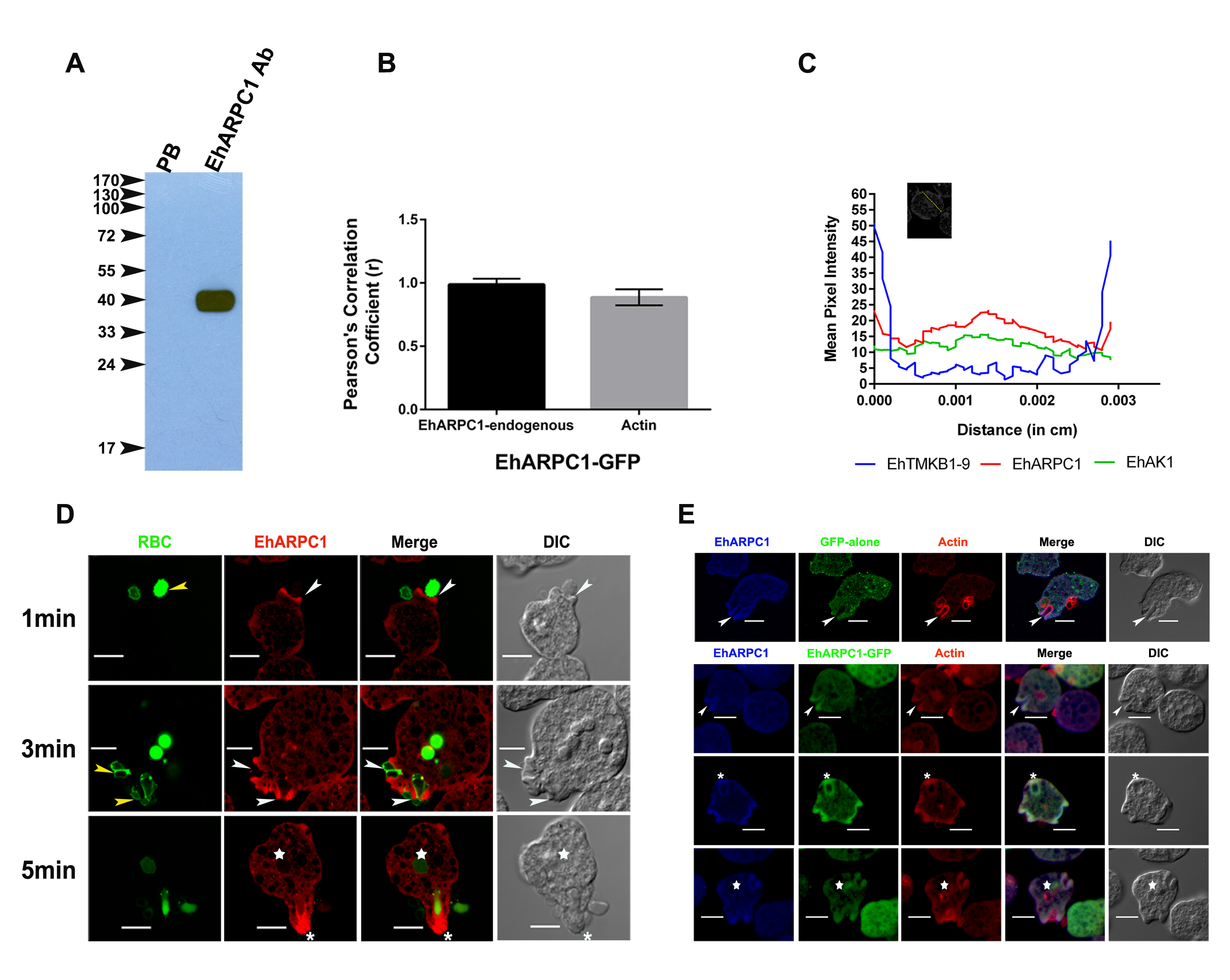

Supplement: S2 Fig — (A) Western blot analysis was performed for checking the specificity of Anti-EhARPC1 antibody raised against recombinant protein. Entamoeba lysate (75μg) was probed with Anti-EhARPC1 (1:1, 000). Pre-bleed was taken as control. Expected size of EhARPC1 is 41 KDa (B) Quantitative analysis of colocalisation of EhARPC1-GFP with endogenous EhARPC1 or Actin was carried out using Pearson’s correlation coefficient (r) using ten stained images. (C) Intensity profiles represent the fluorescence intensity of EhARPC1, EhAK1 and EhTMKB1-9 in cytoplasm and membrane. Results were calculated from ten randomly selected cells using Image J software. Snapshot of ROI (a line across the cell) selected for quantification in a single cell is shown in the box. (D) E. histolytica cells were incubated with CFSE labelled green colored RBC’s for different time points and then cells were fixed and stained for EhARPC1 antibody followed by Alexa 555. (E) E. histolytica cells either expressing GFP alone (top panel) or EhARPC1-GFP (lower panel) were incubated with RBC for 5 min at 37°C. The cells were then fixed and immunostained with anti-EhARPC1 antibody followed by Pacific blue-410. F-actin was stained with TRITC phalloidin and EhARPC1-GFP was immunostained with anti-GFP antibody followed by Alexa 488-labelled secondary antibody. Arrowhead indicate phagocytic cups, asterisk mark just closed cups, star denotes phagosome and yellow color arrowhead mark attached RBC. Scale Bar represents 10 μm. (TIF) [file ppat.1005310.s002.tif]

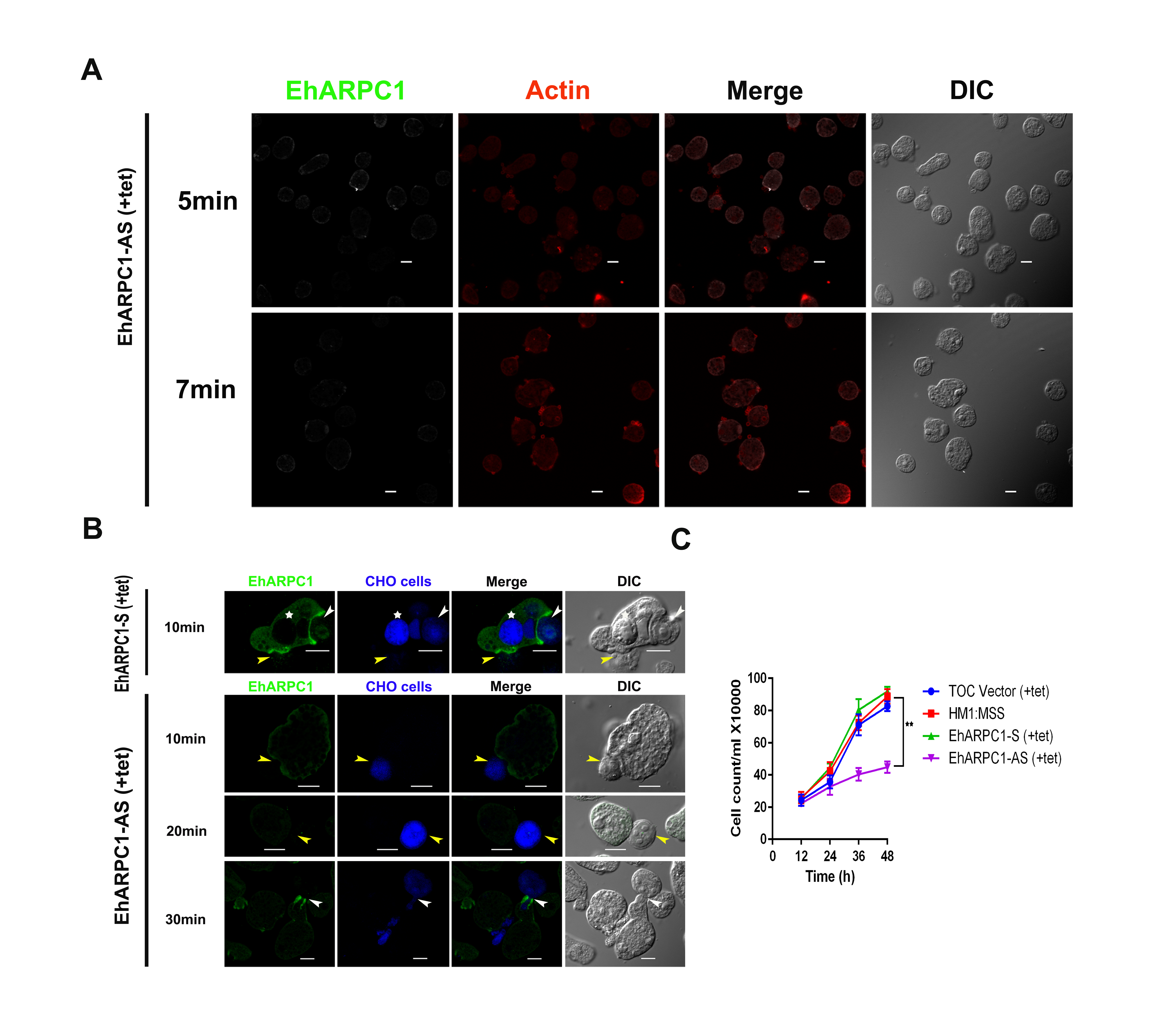

Supplement: S3 Fig — (A) E. histolytica trophozoites expressing anti sense EhARPC1 RNA were incubated with RBC for indicated time interval (3, 5 and 7 min) at 37°C. The cells were then fixed and immunostained with EhARPC1 antibody followed by Alexa 488. Actin was stained with TRITC-phalloidin. Green color is pseudo-colored to gray for efficiently showing the low fluorescent signals from EhARPC1-AS cell line. (B) Cells overexpressing either sense or antisense constructs of EhARPC1 were incubated with cell tracker blue dye-labelled live CHO cells for the indicated times at 37°C. Cells were then fixed and stained for EhARPC1 followed by Alexa-488conjugated secondary antibody. Phagocytic cups are marked by arrowhead, star marks just closed cup and yellow arrowhead show attached CHO cells at the site of phagocytosis. (C) Proliferation of E. histolytica trophozoites carrying different constructs was studied. All cells were grown in presence of 10 μg/ml hygromycin and tetracycline was added to the medium at 30 μg/ml at 0 h. Cells were grown in 5 ml culture tubes in triplicate for all the experiments and counting was carried out using a haemocytometer, after chilling the tube for 5 min. One-way ANOVA test was used for statistical comparisons. “Two black star”p-value≤0.005. (TIF) [file ppat.1005310.s003.tif]

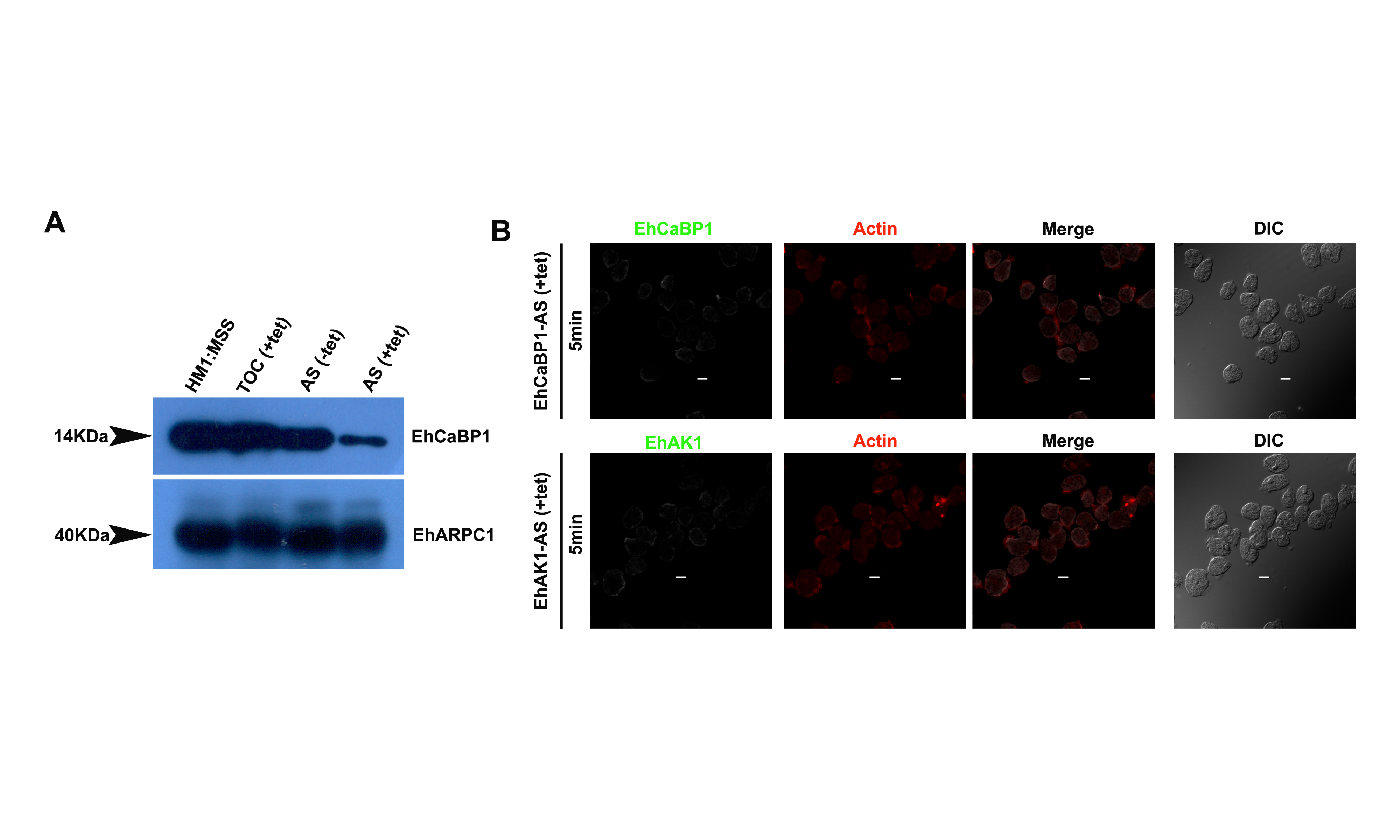

Supplement: S4 Fig — (A) Western blot analysis of amoebic cells expressing antisense EhCaBP1 RNA showing the level of EhCaBP1 and EhARPC1 in tet-inducible vector alone, antisense EhCaBP1 (AS in the presence and the absence of tetracycline (30μg/ml). EhARPC1 was used as an internal control. TOC is tet-o-CAT vector. (B) E. histolytica trophozoites expressing either anti sense EhCaBP1 or EhAK1 RNA were incubated with RBC for 5min time interval at 37°C. The cells were then fixed and immunostained with EhCaBP1 or EhAK1 antibody followed by Alexa 488. Actin was stained with TRITC-phalloidin. Green color is pseudo-colored to gray for efficiently showing the low fluorescent signals from EhCaBP1-AS and EhAK1-AS cell lines. (TIF) [file ppat.1005310.s004.tif]

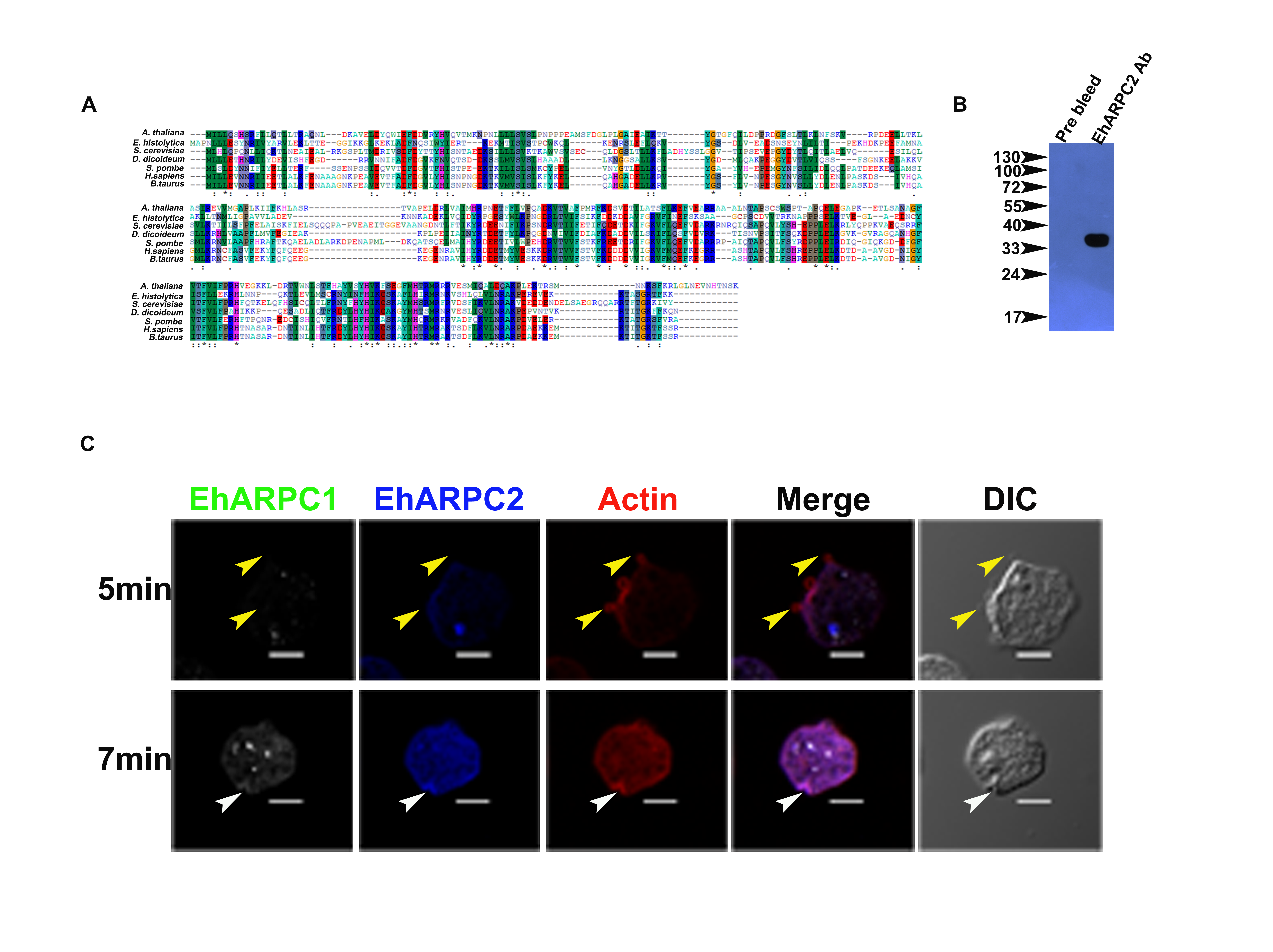

Supplement: S5 Fig — (A) Sequence alignment of E. histolytica ARPC2 with Arp2/3 complex subunit 2 from Saccharomyces cerevisiae, Dictyostelium discoideum, Homo sapiens, Bostarus, Schizosachromyces pombe, Arabidopsis thaliana. (B) Western blot analysis was performed for checking the specificity of Anti-EhARPC2 antibody raised against recombinant protein. Entamoeba lysate (100μg) was probed with Anti-EhARPC2 (1:1, 000). Pre-bleed was taken as control. Expected size of EhARPC2 is 34 KDa. (C) For immunostaining, amoebic cells containing EhARPC1 antisense construct in which cells were grown for 48 h in the presence of 30μg/ml tet were incubated with RBCs for indicated times at 37°C. The cells were then fixed and immunostained with anti-EhARPC1 and anti-EhARPC2 antibody as indicated and double stained with Pacific blue-410 and Alexa 488-labelled secondary antibodies respectively. F-actin was stained with TRITC-phalloidin. Yellow arrowhead shows the site of RBC attachment. Scale Bar represents 10 μm. (TIF) [file ppat.1005310.s005.tif]
