# Supplementary material for: The Entamoeba histolytica, Arp2/3 Complex Is Recruited to Phagocytic Cups through an Atypical Kinase EhAK1
Source: PLoS Pathog. 2015 Dec 8;11(12):e1005310. doi: 10.1371/journal.ppat.1005310 (PMC4672914; doi:10.1371/journal.ppat.1005310)
Supplement: S1 Table — (DOCX) [file ppat.1005310.s011.docx]

Table S1: Actin binding proteins involved in cytoskeleton dynamics (and phagocytosis) in *E. histolytica*.*

| Name of Proteins involved in actin dynamics | Function^#^ | Bioinformatic analysis** | Mass spectrometry analysis of Phagosomes*** | Function involvement in *E. histolytica* |
| --- | --- | --- | --- | --- |
| 1) Arp2/3 complex  Arp2  Arp3 ARPC1 ARPC2  ARPC3 ARPC4 ARPC5 | Nucleation of pre-existing actin filaments which leads to formation of “y” shaped branches [1]. | +  +  +  +  +  +  + | +  +  +  +  -  +  - | Arp2 of D. discoideum can be genetically complemented with Arp2 of *E.histolytica* [2]. Arp3 is shown to be present at the phagocytic cups [3]. |
| 2) Formins   - Formin1 - Formin2 - Formin3 - Formin4 - Formin5 - Formin6 - Formin 7 - Formin 8 | It nucleates actin filaments and forms unbranched filaments. Also it binds to the barbed end of actin filaments and prevents binding of capping proteins [4]. | +  +  +  +  +  +  +  + | +  +  +  -  -  -  -  - | EhFormin 1 is involved in actin polymerization and EhRho1 stimulates its activity [5].  EhFormin 1 and 2 are involved in amoebic motility, phagocytosis and pinocytosis [6]. |
| 3) WASP/Scar | It activates Arp2/3 complex via its verprolin-homology [V] domain, cofilin-homology [C] domain and an acidic [A] region present at the C-terminal [7]. | N/I | N/I | N/I |
| 4) WASH | WASH is a new family member of WASP proteins. It is involved in Arp2/3 activation and actin polymerization [8]. | + | - | N/D |
| 5) MIM | It belongs to I-BAR family of proteins which binds to phosphoinositide-rich membranes and generates negative curvature. It couples actin dynamics to plasma membrane [9]. | + | - | N/D |
| 6) Gelsolin | It severs and caps actin filaments in a Ca^2+^ dependent manner [10]. | + | - | EhABPH is the only gelsolin related protein characterized. It has N-terminal coronin like domain followed by gelsolin/villin like domain [11]. |
| 7) Villin | It comprises of actin binding domain at C-terminal, known as head piece in addition to gelsolin homology domain. It is involved in capping, severing and bundling of actin filaments [12]. | + | + | N/D |
| 8) Flightless I | It belongs to gelsolin family of actin binding proteins. It possess Leucine rich repeats at the N-terminal region and is involved in actin cytoskeleton rearrangements [13]. | + | - | *E. histolytica* Villidin lacks a WD40 domain and shows greater similarity to flightless I than to villidin [14]. |
| 9) Villidin | It is a multidomain protein which not only comprises of gelsolin homology domain and headpiece like villin but also WD repeats and PH domain. It links membrane with the actin dynamics and have potential sites for actin binding in N-terminal region [15]. | + | + | EhABPH is the only gelsolin related protein characterized in Entamoeba and is orthologue of villidin [11]. |
| 10) Coronin | Coronin is involved in actin dynamics in two ways. First at the at the front end of actin dynamics,it protects ATP- bound filaments from cofilin thus preventing premature disassembly and also recruits Arp2/3 complex to filament sides. At the rear ends it works in cooperation with cofilin and dismantles ADP-actin filaments [16]. | + | + | Only EhABPH is shown to have N-terminal coronin like domain followed by gelsolin/villin like domain [11]. |
| 11) Actophorin | It is an ADF/cofilin family member. It severs actin filaments in a dose dependent manner and sequesters actin monomers [17]. | - | + | N/D |
| 12) ADF/Cofilin | ADF is actin depolymerizing factor which has higher affinity for ADP-actin-monomers and filaments than  ATP-actin.  It possess concentration dependent effect on actin dynamics. It severs the filament if the ratio of cofilin/actin subunits in a  Filament is low. However at a high cofilin/actin ratio, it stabilizes the F-actin in a twisted form [18]. | - | + | No studies are done in *E. histolytica* but the role of actin depolymerizing factor [ADF]/cofilin [Cfl] family proteins have shown in *E. invadens* with respect to encystation and excystation [19]. |
| 13) Coactosin | It is an ADF/cofilin family member which binds to F-actin and causes severing of filaments [18]. | - | + | EhCoactosin binds to both G-actin and F-actin. In comparison to Coactosin-like protein it stabilizes the actin filaments and inhibits phagocytosis when over expressed in Entamoeba trophozoites [20]. |
| 14) Twinfilin | It comprises of two ADF homology domain. It forms an equal stoichiometry complex with ADP-actin-monomers, thus preventing any nucleotide exchange which in turns inhibit the assembly of the monomers into the filament [21]. | - | - | N/D |
| 15) CAP | CAP is a cyclase associated protein, it sequester G-actin monomer and inhibits actin polymerization [22]. | - | + | N/D |
| 16) Profilin | It sequesters actin monomers in a 1:1 complex and releases actin on increasing concentration of phosphatidylinositol [4,5]-bisphosphate [23]. | - | + | Studies on *E. histolytica* profilin have shown that it interacts with both rabbit muscle actin and *A.thaliana* actin [24]. |
| 17] α-actinin  α-actinin 1  α- actinin 2 | It comprises of actin binding domain, rod domain and CaM like domain. It helps in cross-linking actin filaments which leads to formation of actin meshwork [25]. | + | + | Both α-actinin1 and α-actinin2 proteins have similar domain organization expect the rod domain which is shorter than the rod domain in α-actinins of higher organisms. Both the protein binds and cross-links actin filaments in a calcium-dependent manner [26, 27]. |
| 18) Filamin | Filamins forms orthogonal F-actin networks and stabilizes and cross link cortical actin to cellular membranes [28]. | + | + | EhFLN [previously known as EhABP-120], a filamin protein is recruited at the plasma membrane via PI[3]P and phosphatidic acid [PA]. When the d100 region of EhFLN required for binding to PA, was over-expressed it increased the amoebic motility suggesting its role in actin dynamics.  The EhABP-120 carboxy-terminal domain [END] was able to associate to 3-sulfate galactosyl ceramide, a new lipid target for a member of the filamin family [29,30]. |
| 19) Myosins IB | It is a group of actin binding proteins that causes contraction of actin filaments by hydrolysis of ATP. It comprises of myosin heavy chain [mhc] and a regulatory light chain. Heavy chain possess ATPase and actin binding domain [31]. | + | + | Myosin IB is involved in erythrophagocytosis and it accumulates both at the phagocytic cup and phagosomes and its tail interacts with F-actin. It also localizes at the pseudopods, vesicles and underneath the plasma membrane [32-34]. Recent studies have also shown the interaction of Myosin 1B with Calmodulin-like calcium binding protein EhCaBP3 in a Ca^2+^ dependent manner [35]. |
| 20) Actobindin | It is an actin monomer binding protein which inhibits actin polymerization [36, 37]. | - | + | N/D |
| 21) Cortexillin | Cortexillins are F-actin bundling proteins that preferentially forms anti-parallel bundles of actin [38]. | - | + | N/D |
| 22) Talin [also called Filopodin] | In motile cell protrusions, Talin plays an important role where it nucleates actin filaments. It binds to vinculin, integrins and links the actin filaments with the plasma membrane [39]. | - | + | N/D |

*****Summary of all information collected from either bioinformatics analysis or phagosome proteome analysis. Experimental identification of protein that participated has also been included.

# Potential function based on results from other systems.

** Bioinformatic analysis data was retrieved from reference; [40]

*** Phagosome proteome data was retrieved from the following references; [3, 41-44].

N/I is Not Identified in *E. histolytica* genome.

N/D is No experimental data in support of the putative function is available.
