# Supplementary material for: The Entamoeba histolytica, Arp2/3 Complex Is Recruited to Phagocytic Cups through an Atypical Kinase EhAK1
Source: PLoS Pathog. 2015 Dec 8;11(12):e1005310. doi: 10.1371/journal.ppat.1005310 (PMC4672914; doi:10.1371/journal.ppat.1005310)
Supplement: S2 Table — (DOCX) [file ppat.1005310.s012.docx]

Table S2: Table summarizing the percentage identity of EhArp2/3 and other actin binding proteins with Arp2/3 complex and actin binding proteins from other organism^#^.

| Name of Protein | *D. discoideum* | *S. cerevisiae* | *S. pombe* | *H. sapiens* | *B. tarus* | *A. thaliana* |
| --- | --- | --- | --- | --- | --- | --- |
| Arp2  (EHI_111050) | 63.64 | 56.36 | 58.59 | 58.18 | 58.18 | 50.39 |
| Arp3  (EHI_198930) | 62.35 | 53.69 | 54.98 | 57.66 | 57.66 | 54.28 |
| ARPC1  (EHI_094120) | 43.10 | 32.86 | 31.74 | 41.79 | 41.79 | 36.97 |
| ARPC2  (EHI_199690) | 31.32 | 31.10 | 31.47 | 34.04 | 34.04 | 22.58 |
| ARPC3  (EHI_172960) | 39.18 | 30.99 | 35.67 | 42.35 | 42.35 | 37.87 |
| ARPC4  (EHI_030820) | 59.17 | 49.41 | 50.60 | 60.71 | 60.71 | 55.03 |
| ARPC5  (EHI_103440) | 28.80 | 16.41 | 20.93 | 22.58 | 22.58 | 22.86 |
| Formin1 (EHI_125300) | 26% (Formin A) |  |  | 27% (mDia 1) |  |  |
| Formin2  (EHI_118260) | 25% (Formin B) |  |  | 30% (mDia 2) |  |  |
| Formin3  (EHI_192460) | 30% (Formin A) |  |  | 25% (Formin 3) |  |  |
| Formin4  (EHI_197550) | 29% (Formin C) |  |  | 22% (mDia 1) |  |  |
| Formin5  (EHI_126070) | 33% (Formin C) |  |  | 20% (mDia 1) |  |  |
| Formin6  (EHI_024650) | 17% (Formin C) |  |  | 23% (mDia 3) |  |  |
| Formin 7  (EHI_197230) | 26% (Formin C) |  |  | 42% (Formin 1) |  |  |
| Formin 8  (EHI_090250) | 32% (Formin C) |  |  | No match found |  |  |
| WASH  (EHI_048670) | 57% with WH2 domain containing protein |  |  | 45% with Wiskott-aldrich syndrome protein (WIPF-1) |  |  |
| MIM  (EHI_016130) | 36% identity with WASP related protein |  |  | 37% identity with unnamed protein product |  |  |
| Gelsolin  (EHI_009570) | 25% identity with GRP-125 (Gelsolin repeated protein) |  |  | 23% identity with Protein flightless-1 homolog |  |  |
| Villin  (EHI_007480) | 28% identity with villin |  |  | 27% identity with Villin-1 |  |  |
| Flightless I  (EHI_150430) | 28% identity with villidin |  |  | 27% identity with villin-1 |  |  |
| Villidin  (EHI_122800) | 26% identity with Villidin |  |  | 24% identity with advillin |  |  |
| Coronin  (EHI_083590) | 50% identity with Coronin |  |  | 39% identity with Coronin 6 |  |  |
| Actophorin  EHI_197480 | 37% identity with hypothetical protein |  |  | 29% identity with destrin isoform a |  |  |
| ADF/Cofilin  (EHI_152990) | 36% with hypothetical protein |  |  | 37% identity with Glia maturation factor |  |  |
| Coactosin  (EHI_168340) | 40% identity with actin binding protein |  |  | 40% identity with Coactosin-like protein |  |  |
| Twinfilin  (EHI_186770) | 39% identity with hypothetical protein |  |  | 33% identity with Twinfilin-1 |  |  |
| CAP  (EHI_136150) | 35% identity with cyclase associated protein |  |  | 31% identity with adenylyl-cyclase associated protein 1 |  |  |
| Profilin  (EHI_176140) | 39% identity with Profilin 1 |  |  | 28% identity with Profilin 2 in PSI-BLAST |  |  |
| α-actinin 1  (EHI_164430) | 24% identity with actin bundling protein |  |  | 32% with α–actinin 1 |  |  |
| α- actinin 2 (EHI_199000) | 45% identity with alpha actinin |  |  | 42% identity with α–actinin 2 |  |  |
| Filamin  (EHI_104630) | 34% identity with gelation factor |  |  | 32% identity with Filamin A |  |  |
| Myosins IB  (EHI_110810) | 55% identity with Myosin IB |  |  | 47% identity with unconventional myosin 1f |  |  |
| Actobindin  (EHI_158570) | 56% identity with hypothetical protein |  |  | 55% identity with SH3 domain interacting protein |  |  |
| Cortexillin  (EHI_104560) | 38% identity to cortexillin II |  |  | Cortexillin is not found in mammalian systems |  |  |
| Talin (also called Filopodin)  (EHI_167130) | 29% identity to Talin homologue |  |  | 27% identity with Talin |  |  |

#- For the percentage identity, p-BLAST was performed using the amoebic protein sequence against different organisms database.
