# Supplementary material for: The Entamoeba histolytica, Arp2/3 Complex Is Recruited to Phagocytic Cups through an Atypical Kinase EhAK1
Source: PLoS Pathog. 2015 Dec 8;11(12):e1005310. doi: 10.1371/journal.ppat.1005310 (PMC4672914; doi:10.1371/journal.ppat.1005310)
Supplement: S3 Table — (DOCX) [file ppat.1005310.s013.docx]

Table S3: List of antibodies used in the study.

| Antigen | Reference |
| --- | --- |
| EhTMKB1-9 | Shrimal, S. *et. al*[45] |
| EhC2PK | Somlata *et.al*[44] |
| EhCaBP1 | Sahoo, N. et al.[46] |
| EhCaBP2 | Sahoo, N. et al.[46] |
| EhCaBP3 | Aslam, S. *et.a*l[35] |
| EhAK1 | Mansuri, M. S. *et. al* [41] |
| EhARPC1 | This study |
| EhARPC2 | This study |
| Poly-His | Sigma |
