# Supplementary material for: The Entamoeba histolytica, Arp2/3 Complex Is Recruited to Phagocytic Cups through an Atypical Kinase EhAK1
Source: PLoS Pathog. 2015 Dec 8;11(12):e1005310. doi: 10.1371/journal.ppat.1005310 (PMC4672914; doi:10.1371/journal.ppat.1005310)
Supplement: S1 References — (DOCX) [file ppat.1005310.s014.docx]

Supplementary References:

1. Borths EL, Welch MD. Turning on the Arp2/3 complex at atomic resolution. Structure. 2002;10(2):131-5. Epub 2002/02/13. PubMed PMID: 11839297.

2. Zaki M, King J, Futterer K, Insall RH. Replacement of the essential Dictyostelium Arp2 gene by its Entamoeba homologue using parasexual genetics. BMC genetics. 2007;8:28. Epub 2007/06/08. doi: 10.1186/1471-2156-8-28. PubMed PMID: 17553170; PubMed Central PMCID: PMCPmc1904233.

3. Marion S, Laurent C, Guillen N. Signalization and cytoskeleton activity through myosin IB during the early steps of phagocytosis in Entamoeba histolytica: a proteomic approach. Cellular microbiology. 2005;7(10):1504-18. doi: 10.1111/j.1462-5822.2005.00573.x. PubMed PMID: 16153248.

4. Goode BL, Eck MJ. Mechanism and function of formins in the control of actin assembly. Annual review of biochemistry. 2007;76:593-627. Epub 2007/03/22. doi: 10.1146/annurev.biochem.75.103004.142647. PubMed PMID: 17373907.

5. Bosch DE, Yang B, Siderovski DP. Entamoeba histolytica Rho1 regulates actin polymerization through a divergent, diaphanous-related formin. Biochemistry. 2012;51(44):8791-801. doi: 10.1021/bi300954g. PubMed PMID: 23050667; PubMed Central PMCID: PMC3491106.

6. Majumder S, Lohia A. Entamoeba histolytica encodes unique formins, a subset of which regulates DNA content and cell division. Infection and immunity. 2008;76(6):2368-78. doi: 10.1128/IAI.01449-07. PubMed PMID: 18347041; PubMed Central PMCID: PMC2423068.

7. Veltman DM, Insall RH. WASP family proteins: their evolution and its physiological implications. Molecular biology of the cell. 2010;21(16):2880-93. Epub 2010/06/25. doi: 10.1091/mbc.E10-04-0372. PubMed PMID: 20573979; PubMed Central PMCID: PMCPmc2921111.

8. Linardopoulou EV, Parghi SS, Friedman C, Osborn GE, Parkhurst SM, Trask BJ. Human subtelomeric WASH genes encode a new subclass of the WASP family. PLoS genetics. 2007;3(12):e237. Epub 2007/12/28. doi: 10.1371/journal.pgen.0030237. PubMed PMID: 18159949; PubMed Central PMCID: PMCPmc2151093.

9. Zhao H, Pykalainen A, Lappalainen P. I-BAR domain proteins: linking actin and plasma membrane dynamics. Current opinion in cell biology. 2011;23(1):14-21. Epub 2010/11/26. doi: 10.1016/j.ceb.2010.10.005. PubMed PMID: 21093245.

10. Sun HQ, Yamamoto M, Mejillano M, Yin HL. Gelsolin, a multifunctional actin regulatory protein. The Journal of biological chemistry. 1999;274(47):33179-82. Epub 1999/11/24. PubMed PMID: 10559185.

11. Ebert F, Guillen N, Leippe M, Tannich E. Molecular cloning and cellular localization of an unusual bipartite Entamoeba histolytica polypeptide with similarity to actin binding proteins. Molecular and biochemical parasitology. 2000;111(2):459-64. Epub 2001/02/13. PubMed PMID: 11163453.

12. Khurana S, George SP. Regulation of cell structure and function by actin-binding proteins: villin's perspective. FEBS letters. 2008;582(14):2128-39. Epub 2008/03/01. doi: 10.1016/j.febslet.2008.02.040. PubMed PMID: 18307996; PubMed Central PMCID: PMCPmc2680319.

13. Davy DA, Campbell HD, Fountain S, de Jong D, Crouch MF. The flightless I protein colocalizes with actin- and microtubule-based structures in motile Swiss 3T3 fibroblasts: evidence for the involvement of PI 3-kinase and Ras-related small GTPases. Journal of cell science. 2001;114(Pt 3):549-62. Epub 2001/02/15. PubMed PMID: 11171324.

14. Ghoshdastider U, Popp D, Burtnick LD, Robinson RC. The expanding superfamily of gelsolin homology domain proteins. Cytoskeleton. 2013;70(11):775-95. Epub 2013/10/25. doi: 10.1002/cm.21149. PubMed PMID: 24155256.

15. Gloss A, Rivero F, Khaire N, Muller R, Loomis WF, Schleicher M, et al. Villidin, a novel WD-repeat and villin-related protein from Dictyostelium, is associated with membranes and the cytoskeleton. Molecular biology of the cell. 2003;14(7):2716-27. Epub 2003/07/15. doi: 10.1091/mbc.E02-12-0827. PubMed PMID: 12857859; PubMed Central PMCID: PMCPmc165671.

16. Gandhi M, Goode BL. Coronin: the double-edged sword of actin dynamics. Sub-cellular biochemistry. 2008;48:72-87. Epub 2008/10/18. doi: 10.1007/978-0-387-09595-0_7. PubMed PMID: 18925372.

17. Maciver SK, Zot HG, Pollard TD. Characterization of actin filament severing by actophorin from Acanthamoeba castellanii. The Journal of cell biology. 1991;115(6):1611-20. Epub 1991/12/01. PubMed PMID: 1757465; PubMed Central PMCID: PMCPmc2289216.

18. Bernstein BW, Bamburg JR. ADF/cofilin: a functional node in cell biology. Trends Cell Biol. 2010;20(4):187-95. doi: 10.1016/j.tcb.2010.01.001. PubMed PMID: 20133134; PubMed Central PMCID: PMCPMC2849908.

19. Makioka A, Kumagai M, Hiranuka K, Kobayashi S, Takeuchi T. Entamoeba invadens: identification of ADF/cofilin and their expression analysis in relation to encystation and excystation. Experimental parasitology. 2011;127(1):195-201. Epub 2010/08/10. doi: 10.1016/j.exppara.2010.07.018. PubMed PMID: 20691182.

20. Kumar N, Somlata, Mazumder M, Dutta P, Maiti S, Gourinath S. EhCoactosin stabilizes actin filaments in the protist parasite Entamoeba histolytica. PLoS pathogens. 2014;10(9):e1004362. doi: 10.1371/journal.ppat.1004362. PubMed PMID: 25210743; PubMed Central PMCID: PMC4161475.

21. Palmgren S, Vartiainen M, Lappalainen P. Twinfilin, a molecular mailman for actin monomers. Journal of cell science. 2002;115(Pt 5):881-6. Epub 2002/03/01. PubMed PMID: 11870207.

22. Hubberstey AV, Mottillo EP. Cyclase-associated proteins: CAPacity for linking signal transduction and actin polymerization. FASEB journal : official publication of the Federation of American Societies for Experimental Biology. 2002;16(6):487-99. Epub 2002/03/29. PubMed PMID: 11919151.

23. Witke W. The role of profilin complexes in cell motility and other cellular processes. Trends Cell Biol. 2004;14(8):461-9. Epub 2004/08/17. doi: 10.1016/j.tcb.2004.07.003. PubMed PMID: 15308213.

24. Binder M, Ortner S, Erben H, Scheiner O, Wiedermann G, Valenta R, et al. The basic isoform of profilin in pathogenic Entamoeba histolytica. cDNA cloning, heterologous expression, and actin-binding properties. European journal of biochemistry / FEBS. 1995;233(3):976-81. Epub 1995/11/01. PubMed PMID: 8521867.

25. Sjoblom B, Salmazo A, Djinovic-Carugo K. Alpha-actinin structure and regulation. Cellular and molecular life sciences : CMLS. 2008;65(17):2688-701. Epub 2008/05/20. doi: 10.1007/s00018-008-8080-8. PubMed PMID: 18488141.

26. Virel A, Addario B, Backman L. Characterization of Entamoeba histolytica alpha-actinin2. Molecular and biochemical parasitology. 2007;154(1):82-9. Epub 2007/06/01. doi: 10.1016/j.molbiopara.2007.04.010. PubMed PMID: 17537529.

27. Virel A, Backman L. Characterization of Entamoeba histolytica alpha-actinin. Molecular and biochemical parasitology. 2006;145(1):11-7. Epub 2005/10/13. doi: 10.1016/j.molbiopara.2005.09.003. PubMed PMID: 16219372.

28. Zhou AX, Hartwig JH, Akyurek LM. Filamins in cell signaling, transcription and organ development. Trends Cell Biol. 2010;20(2):113-23. Epub 2010/01/12. doi: 10.1016/j.tcb.2009.12.001. PubMed PMID: 20061151.

29. Diaz-Valencia JD, Almaraz-Barrera Mde J, Jay D, Hernandez-Cuevas NA, Garcia E, Gonzalez-De la Rosa CH, et al. Novel structural and functional findings of the ehFLN protein from Entamoeba histolytica. Cell motility and the cytoskeleton. 2007;64(11):880-96. Epub 2007/08/21. doi: 10.1002/cm.20232. PubMed PMID: 17705278.

30. Diaz-Valencia JD, Almaraz-Barrera Mde J, Arias-Romero LE, Hernandez-Rivas R, Rojo-Dominguez A, Guillen N, et al. The ABP-120 C-end region from Entamoeba histolytica interacts with sulfatide, a new lipid target. Biochemical and biophysical research communications. 2005;338(3):1527-36. Epub 2005/11/09. doi: 10.1016/j.bbrc.2005.10.119. PubMed PMID: 16274663.

31. Hartman MA, Spudich JA. The myosin superfamily at a glance. Journal of cell science. 2012;125(Pt 7):1627-32. Epub 2012/05/09. doi: 10.1242/jcs.094300. PubMed PMID: 22566666; PubMed Central PMCID: PMCPmc3346823.

32. Marion S, Wilhelm C, Voigt H, Bacri JC, Guillen N. Overexpression of myosin IB in living Entamoeba histolytica enhances cytoplasm viscosity and reduces phagocytosis. Journal of cell science. 2004;117(Pt 15):3271-9. Epub 2004/07/01. doi: 10.1242/jcs.01178. PubMed PMID: 15226399.

33. Vargas MA, Voigt H, Sansonetti P, Guillen N. The tail domain of Entamoeba histolytica myosin IB bind F-actin. Archives of medical research. 1997;28 Spec No:137-8. Epub 1997/01/01. PubMed PMID: 9033044.

34. Voigt H, Olivo JC, Sansonetti P, Guillen N. Myosin IB from Entamoeba histolytica is involved in phagocytosis of human erythrocytes. Journal of cell science. 1999;112 ( Pt 8):1191-201. Epub 1999/03/23. PubMed PMID: 10085254.

35. Aslam S, Bhattacharya S, Bhattacharya A. The Calmodulin-like calcium binding protein EhCaBP3 of Entamoeba histolytica regulates phagocytosis and is involved in actin dynamics. PLoS pathogens. 2012;8(12):e1003055. doi: 10.1371/journal.ppat.1003055. PubMed PMID: 23300437; PubMed Central PMCID: PMC3531509.

36. Bubb MR, Knutson JR, Porter DK, Korn ED. Actobindin induces the accumulation of actin dimers that neither nucleate polymerization nor self-associate. The Journal of biological chemistry. 1994;269(41):25592-7. Epub 1994/10/14. PubMed PMID: 7929262.

37. Bubb MR, Lewis MS, Korn ED. Actobindin binds with high affinity to a covalently cross-linked actin dimer. The Journal of biological chemistry. 1994;269(41):25587-91. Epub 1994/10/14. PubMed PMID: 7929261.

38. Cha I, Jeon TJ. Dynamic localization of the actin-bundling protein cortexillin I during cell migration. Molecules and cells. 2011;32(3):281-7. Epub 2011/06/29. doi: 10.1007/s10059-011-0072-0. PubMed PMID: 21710202; PubMed Central PMCID: PMCPmc3887633.

39. Critchley DR, Gingras AR. Talin at a glance. Journal of cell science. 2008;121(Pt 9):1345-7. Epub 2008/04/25. doi: 10.1242/jcs.018085. PubMed PMID: 18434644.

40. Hon C.C. et al. Dissecting the Actin cytoskeleton of Entamoeba histolytica from a genomic perspective. In: C. Graham Clark, Patricia J. Johnson and Rodney D. Adam editors. Anaerobic Parasitic Protozoa: Genomics and Molecular Biology. Caister Academic Press; 2010. pp. 81-118. PMID:17497115.

41. Mansuri MS, Bhattacharya S, Bhattacharya A. A novel alpha kinase EhAK1 phosphorylates actin and regulates phagocytosis in Entamoeba histolytica. PLoS pathogens. 2014;10(10):e1004411. doi: 10.1371/journal.ppat.1004411. PubMed PMID: 25299184; PubMed Central PMCID: PMC4192601.

42. Marion S, Guillen N. Genomic and proteomic approaches highlight phagocytosis of living and apoptotic human cells by the parasite Entamoeba histolytica. International journal for parasitology. 2006;36(2):131-9. doi: 10.1016/j.ijpara.2005.10.007. PubMed PMID: 16386742.

43. Okada M, Nozaki T. New insights into molecular mechanisms of phagocytosis in Entamoeba histolytica by proteomic analysis. Archives of medical research. 2006;37(2):244-52. doi: 10.1016/j.arcmed.2005.10.003. PubMed PMID: 16380325.

44. Somlata, Bhattacharya S, Bhattacharya A. A C2 domain protein kinase initiates phagocytosis in the protozoan parasite Entamoeba histolytica. Nature communications. 2011;2:230. doi: 10.1038/ncomms1199. PubMed PMID: 21407196.

45. Shrimal S, Bhattacharya S, Bhattacharya A. Serum-dependent selective expression of EhTMKB1-9, a member of Entamoeba histolytica B1 family of transmembrane kinases. PLoS pathogens. 2010;6(6):e1000929. doi: 10.1371/journal.ppat.1000929. PubMed PMID: 20532220; PubMed Central PMCID: PMC2880585.

46. Sahoo N, Labruyere E, Bhattacharya S, Sen P, Guillen N, Bhattacharya A. Calcium binding protein 1 of the protozoan parasite Entamoeba histolytica interacts with actin and is involved in cytoskeleton dynamics. Journal of cell science. 2004;117(Pt 16):3625-34. doi: 10.1242/jcs.01198. PubMed PMID: 15252130.
